# Limits of Applicability of the Voronoi Tessellation Determined by Centers of Cell Nuclei to Epithelium Morphology

**DOI:** 10.3389/fphys.2016.00551

**Published:** 2016-11-25

**Authors:** Sara Kaliman, Christina Jayachandran, Florian Rehfeldt, Ana-Sunčana Smith

**Affiliations:** ^1^Physics Underlying Life Sciences Group, Institute for Theoretical Physics and Cluster of Excellence: Engineering of Advanced Materials, Friedrich Alexander University Erlangen–NürnbergErlangen, Germany; ^2^Third Institute of Physics–Biophysics, Georg-August-UniversityGöttingen, Germany; ^3^Group for Computational Life Sciences, Division of Physical Chemistry, Institute Ruđer BoškovićZagreb, Croatia

**Keywords:** epithelial tissue morphology, Voronoi tessellation, fluorescence microscopy, nuclei segmentation, image analysis, MDCK, cross-correlations, cell shape

## Abstract

It is well accepted that cells in the tissue can be regarded as tiles tessellating space. A number of approaches were developed to find an appropriate mathematical description of such cell tiling. A particularly useful approach is the so called Voronoi tessellation, built from centers of mass of the cell nuclei (CMVT), which is commonly used for estimating the morphology of cells in epithelial tissues. However, a study providing a statistically sound analysis of this method's accuracy is not available in the literature. We addressed this issue here by comparing a number of morphological measures of the cells, including area, perimeter, and elongation obtained from such a tessellation with identical measures extracted from direct imaging acquired by staining the cell membranes. After analyzing the shapes of 15,000 MDCK II epithelial cells under several conditions, we find that CMVT reasonably well reproduces many of the morphological properties of the tissue with an error that is between 10 and 15%. Moreover, cross-correlations between different morphological measures are reproduced qualitatively correctly by this method. However, all of the properties including the cell perimeters, number of neighbors, and anisotropy measures often suffer from systematic or size dependent errors. These discrepancies originate from the polygonal nature of the tessellation which sets the limits of the applicability of CMVT.

## Introduction

Over the last decade, a global effort to understand the underlying principles of morphogenesis, wound healing and cancer progression has generated a tremendous momentum in studies of epithelial tissues (Zorn et al., 2015). Consequently, significant work to characterize their architecture and growth is being performed on *in vivo* and *in vitro* model systems, a prototypical example of the latter being the MDCK cell monolayers (Trepat et al., 2009; Angelini et al., 2011; Puliafito et al., 2012; Harris et al., 2013; Deforet et al., 2014; Kaliman et al., 2014; Streichan et al., 2014; Das et al., 2015; Zehnder et al., 2015). Such progress is founded on the remarkable advance of molecular biology and imaging techniques, whose output data forms the basis for the quantitative analysis of the tissue development (Ntziachristos, 2010). However, optimally harvesting this data depends on the development of image analysis tools. One commonly used technique for gaining information about the internal tissue organization is based on the construction of appropriate space tessellations.

For epithelial cells, it was suggested already in 1978 that the polygonal Voronoi tessellation (VT) well approximates the tissue structure (Honda, 1978). This prompted the development of *in silico* models, which adopt the polygonal nature of cells and are parametrized to reproduce the distributions of morphological features such as the area and the perimeters of the cells. These models typically use a free energy functional, which is minimized to yield optimal positions of points (Sulsky et al., 1984) (Mkrtchyan et al., 2014) generating the tessellation. Alternatively, vertex models optimize the cell area and the boundary-length between cells. The parameters of the free energy function yield insights into the mechanical state of the tissue (Farhadifar et al., 2007; Hannezo et al., 2014; Dapeng et al., 2015) even though the one-to-one correspondence with cells in acquired images cannot be established.

**Figure 1:**
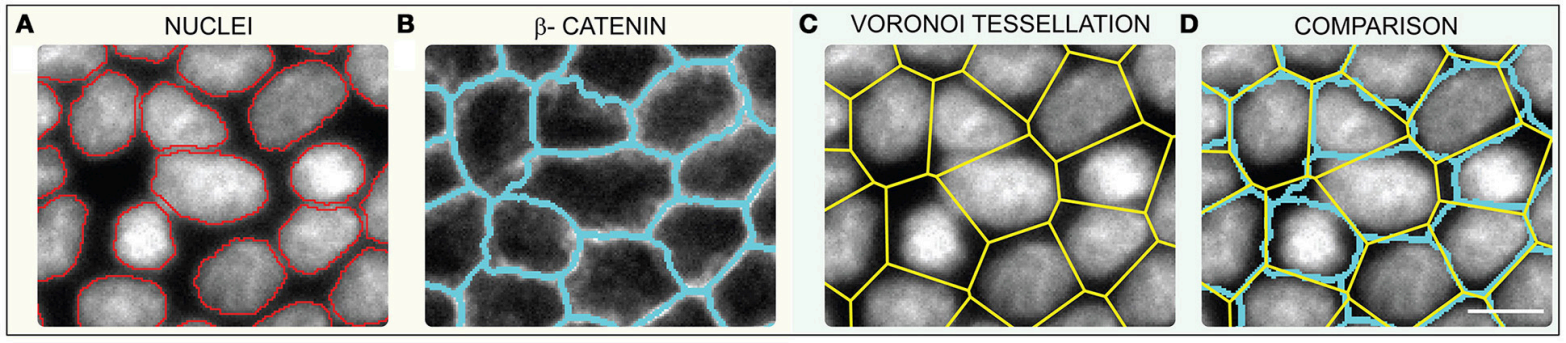
**(A)** Image of cell nuclei (Hoechst stained) and segmented nuclei edges (red) **(B)** Non-processed membrane image (β-catenin) overlaid with the image segmented with a watershed algorithm (blue). CMVT laid over **(C)** cell nuclei and **(D)** the membrane. Scale bar is 10 μm.

Besides modeling, VTs are often applied in direct analysis of fluorescence microscopy data. Tessellations offer simple, fast and fully automated access to tissue morphology, which is otherwise difficult to obtain for a large number of cells. Tessellations are frequently generated from the centers of mass of cell nuclei (CMVT), which themselves are determined from segmented images (Figures 1A–C). Today, CMVT make an integral part of automated image analysis packages used, for example, to delineate cancerous and healthy tissue in histopathological samples. One of the first attempts to use CMVT in a clinically relevant situation was to estimate cell areas and perimeters in primary lung carcinoma (Kayser and Stute, 1989). More accurate and complex procedures developed over time include one using CMVT to characterize a number of morphological measures of cell shapes in different cancers with poor and good prognosis (Sudbø et al., 2000) Recently, CMVT became the foundation of an automatic analysis routine and is today used for the analysis of biopsies to distinguish cervical inter-epithelial neoplasia from normal tissues (Guillaud et al., 2014) (Sheikhzadeh et al., 2015).

For a long time CMVT was occasionally used in studies of reconstituted tissues as part of the effort to elucidate biochemical and physical principles of tissue growth (Zorn et al., 2015). It was applied in an *in-vitro* characterization of the effect of cell medium on the growth of two colorectal cancer cell lines (Darro et al., 1993). With the advance of imaging, CMVT was employed more frequently, due to its simplicity and accessibility of nuclei staining procedures. In recent years it was used to estimate areas of MDCK cells within a monolayer, and to understand fluctuations of the cell volume (Zehnder et al., 2015). Furthermore, it was applied in the quantification of the cell proliferation rate as a function of the cell area (Streichan et al., 2014), and in evaluating the time dependence of the average cell elongation in MDCK colonies (Puliafito et al., 2012).

This wide spread usage of CMVT demonstrates that this technique is becoming an accepted and fairly common method for reconstruction of the cells' shapes. It is therefore surprising that a procedure for quantitative and systematic analysis of this approximation is still lacking in literature. While visual comparison of the tessellation with the images of the cell membrane suggests that CMVT can capture a number of cell shape characteristics at least in some tissues, direct correspondence of reconstructed shapes and the true cell morphology was not validated in a quantitative manner on a statistically sound sample of any system. Actually, the accuracy of CMVT may vary in different tissues and therefore the applicability of the tessellation should be tested whenever CMVT is used, particularly for diagnostic purposes.

In this paper we develop a protocol for the analysis of the accuracy of CMVT and apply this procedure to MDCK monolayers. We show, on a sample of 15,000 MDCK-II cells (cell areas 74–274 μm^2^), generated by imaging 3 day old model-tissues grown on collagen I coated elastic polyacrylamide gels (*E* = 11–34 kPa), that CMVT indeed reasonably captures the cells' shapes. Similar results are obtained for different growth conditions (cells grown on substrates with a Young's modulus of *E* = 0.6 kPa and glass), but with slightly lower statistics. However, due to its intrinsic polygonal nature, CMVT cannot reproduce the curved cell boundaries or avoid cutting through the nucleus interior (Figure 1C). Consequently, while instructive, the correspondence of the CMVT and the original data cannot be significantly improved with better imaging.

To quantify the accuracy of the CMVT we analyze a number of classic shape measures, namely cell area, perimeter, and number of neighbors of each cell. Furthermore, we investigate cell anisotropy measures; cell elongation (ratio of major and minor principle axis of cells), variations in boundary-lengths (deviation of the mean boundary-length that a cell has with each neighbor), and co-alignment between cell body and its nucleus (the angle between major axes of cell and nucleus). To assess the quality of CMVT, we also determine morphological measures directly from fluorescent images of the plasma membrane immuno-stained for β-catenin.

## Experimental materials and methods

### Tissue culture, gel preparation, and fluorescent staining

MDCK II cells were purchased from ECACC (#00062107) and cultured in MEM Earle's medium (Biochrom, #F0325) supplemented with 5% fetal bovine serum (FBS, #F0804, Sigma-Aldrich), 2 mM L-glutamine (Sigma-Aldrich, #G7513), 1% P/S (penicillin, streptomycin) (Gibco, LifeTechnologies, #15070-063) at 37°C and 5% CO_2_. Cells were trypsinized and passaged every 2 or 3 days before reaching 80% confluence.

Elastic poly-acrylamide (PA) gels (*E* = 0.6–34 kPa) were prepared as described earlier (Kaliman et al., 2014). In brief: Mixtures of acrylamide (40% solution, BioRad) and bis-acrylamide (2% solution, BioRad) were polymerized for 60 min on plasma cleaned glass cover slips (No. 1, 25 mm Ø, VWR) that were functionalized with 3-aminoproyltriethoxysiliane (APTES, Sigma-Aldrich, Germany) for 15 min and incubated with a 0.5% solution of glutaraldehyde in PBS (Sigma Aldrich, Germany) for 30 min. The hydrogels were subsequently coated with Collagen-I (BD Biosciences) at 0.02 mg/mL in a 50 mM HEPES buffer using the bi-functional cross-linker Sulfo-SANPAH (Pierce, Thermo Scientific). For quality control, the Young's modulus *E* was measured macroscopically by a cone and plate rheometer (MCR 501, Anton Paar, Austria).

Cells were fixed using a 10% solution of formaldehyde for 5 min, then washed and blocked with a 2% BSA (Sigma Aldrich) solution in PBS, and permeabilized for using a 0.5% solution of Triton X 100 (Carl Roth, Germany). Filamentous actin was stained using phalloidin–tetramethylrhodamine B isothiocyanate (TRITC, #77418-100UG, Sigma-Aldrich), β-catenin with a combination of primary (anti-beta-catenin AB produced in rabbit, Sigma-Aldrich) and secondary antibody (anti rabbit IgG-FITC, Sigma-Aldrich), and the DNA in the nucleus was labeled with Hoechst (#33342, Molecular Probes, Life Technologies).

Following this procedure, we obtain tissues of MDCK cells grown on stiff gels in the density range of about 4800–8600 cells/mm^2^. On glass we explore densities of 4500–5700 cells/mm^2^ while on very soft substrates (*E* = 0.6 kPa) we systematically recover densities of 12,500–13,000 cells/mm^2^.

### Image acquisition

Fluorescence microscopy was done with an inverted microscope (Axio Observer.Z1) using a 20x objective (N-Achroplan) and the HXP 120 illumination source (all from Zeiss, Göttingen). Images were recorded with a Zeiss AxioCam (MRm Rev. 3 FireWire) using the Zeiss AxioVision software package (Rel. 4.7). For the different fluorescence channels, the following acquisition times were used: Hoechst (nucleus) 400 ms, TRITC (actin) 200 ms, and FITC (β-catenin) 500 ms. All images were saved in an uncompressed TIFF format in a resolution of 1388 (H) × 1040 (V) = 1.4 Mega Pixel with 8 bit depth resulting in a pixel length 0.31 μm. Stitching of images is not performed to avoid introducing small shifts, which could affect the evaluation of the morphological properties of cells extracted from such images.

Confocal imaging was performed to provide a deeper understanding of the origins of errors associated with CMVT. Images were acquired with a Leica LSM 5 laser-scanning microscope equipped with a white light laser and a 63x oil immersion objective.

### Image analysis

#### Morphological properties of cell nuclei

Voronoi tessellations are typically obtained from immuno-fluorescent images of cell nuclei. These images need to be segmented with great accuracy to correctly determine all individual objects (nuclei) and their centers of mass. As summarized in a couple of recent reviews (Meijering, 2012) (Xing and Yang, 2016), a number of methods were developed for the segmentation of nuclei images over the last 50 years. One common approach is used when a shape with several concave points appears in the image after thresholding. In this approach, this object is interpreted as two or more merged nuclei (Zhang et al., 2013) (Wienert et al., 2012). However, most common are approaches based on the watershed algorithm (Vincent and Soille, 1991) (Malpica et al., 1997), which is either applied to the original data, or on a distance-transformed binary image (Xing and Yang, 2016). Watershed-based methods are implemented for example in widely used software such as ImageJ and CellProfiler. In most of these methods, difficulties arise when nuclei cover a large fraction of the surface. In most of these cases, the performance can be significantly improved by manual pre-processing of images.

**Figure 2:**
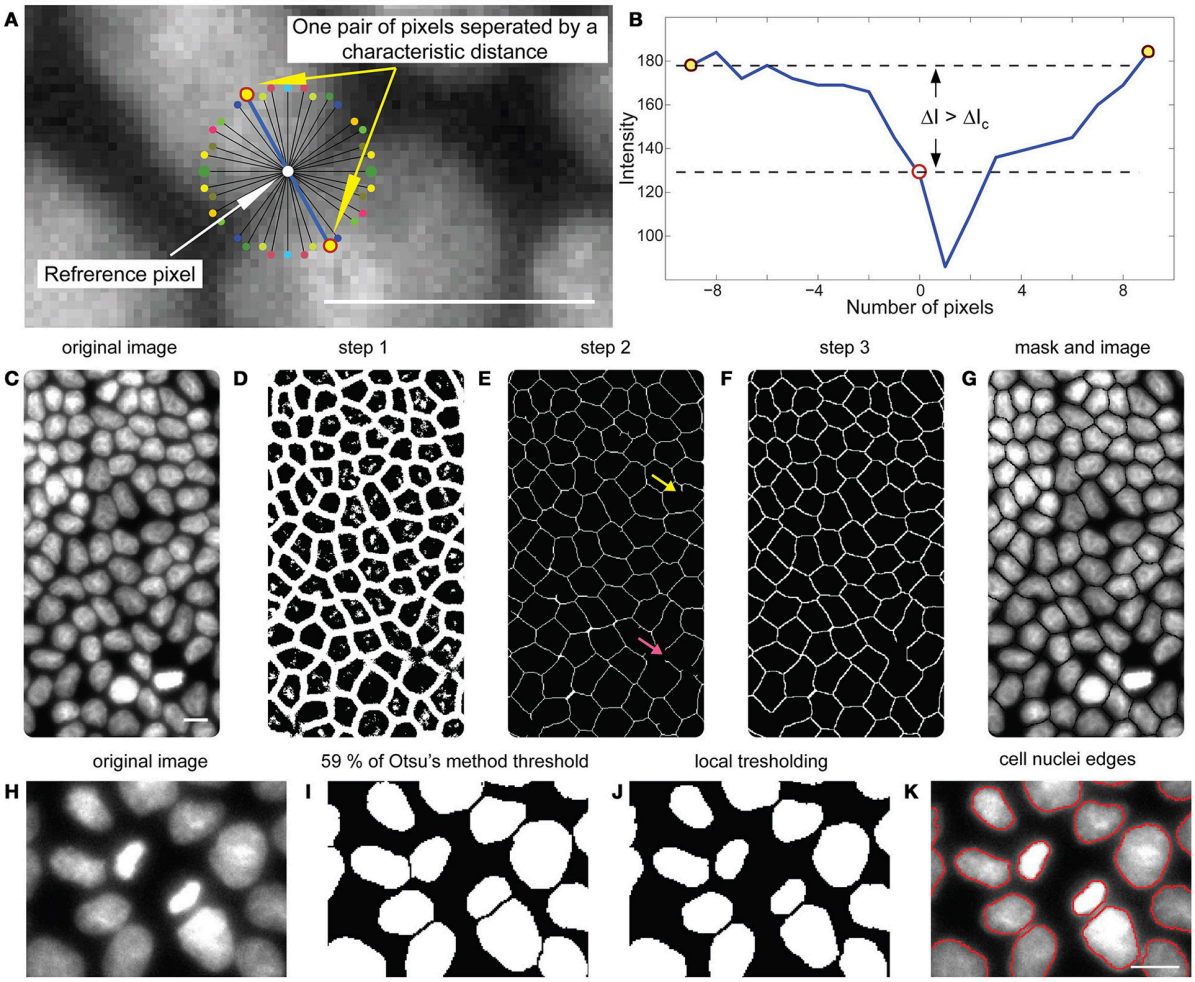
**Nuclei segmentation protocol. (A)** 18 pairs of stencil arms. **(B)** Intensity values along the two arms of the stencil making a par (blue line in the **(A)** panel). In this particular example, there is at least one pair of arms for which the difference in brightness between the referent pixel and the arm ends ΔI is larger than Δ*I*_*c*_, as shown in the graph. Consequently, this pixel contributes to the image of low intensity regions. **(C)** Image of nuclei after contrast increasing. **(D)** Image of low intensity region acquired after the first step in the segmentation procedure. **(E)** Precursor of the mask emerging from the second step of the segmentation procedure. Examples of false and disconnected dangling branches are indicated with yellow and red arrows. **(F)** The completed mask. **(G)** Segmentation mask is superimposed onto the enhanced image before thresholding. **(H)** A region of interest from the original image. **(I)** Result of the first step of the threshold procedure. **(J)** Result of the local thresholding procedure. **(K)** Boundaries of the nuclei (red) extracted from image after thresholding. Scale bars are 10 μm.

We here develop a fully automated procedure optimized for cell monolayers where the cell nuclei do not overlap in principle. The routine (Figure 2) is particularly tuned to recognize boundaries between cell nuclei, hence, avoiding undercounting in the relevant image. Its foundation is a minimum intensity mask that is built around each nucleus, before the local threshold is applied. It is implemented in two stages. The first stage involves building a mask based on local intensity minima (space between cell nuclei) and in the second stage, the mask is superimposed on the original image before a local thresholding is performed. The binary image obtained is used to determine the boundaries of the cells' nuclei. Our fully automated method works with accuracies larger than 99% as determined by manually counting segmentation errors (see Supplementary Section 1).

##### Step 1–finding low intensity regions

Initially, the contrast of the image is increased by linear remapping of the original image intensity range onto the intensity interval [0, 255] (Figure 2C). To determine the mask, the environment of every pixel in the image is tested, by creating a 36 arms of the stencil on the circle of fixed radius *r*, with the reference pixel being in the center (Figure 2A). Radius *r* is the first manually adjusted parameter and depends on magnification and camera resolution used to acquire the images. The arms of the stencil closing 180° are coupled resulting in 18 arm pairs. If extremal pixels of the arms are brighter than the reference pixel by predefined value Δ*I*_*c*_ (Figure 2B), the reference pixel is stored in a 2D array, which is updated at every step. After all pixels in the image are tested the 2D array forms an image of low intensity regions (Figure 2C), roughly representing the space between nuclei. For distinct experimental conditions (staining method, magnification, and camera), *r* and Δ*I*_*c*_ are kept constant for all images (*r* = 9, Δ*I*_*c*_ = 3).

##### Steps 2 and 3–post-processing the image of low intensity regions and creating a mask

In the second step, pixels of low intensity which are not part of the space between nuclei are eliminated by performing a set of morphological operations to the image of low intensity regions. Specifically, a pixel is set to be white (element of the region) if more than five pixels in its 3 × 3 neighborhood are white. Disconnected small white objects are removed from the image. The resulting image is then subject to dilatation, skeletonization, and to the removal of spur pixels, yielding a one pixel thick network (Figure 2E) that is a precursor of the future mask.

The image obtained still suffers from dangling branches in the network, which may be false boundaries (yellow arrow in Figure 2E), or parts of missing boundaries (red arrow). To reconstruct these, a search for a matching dangling branch is performed in the radius of 27 pixels (8.4 μm), around each disconnected end in the image. If a match is found and the extension of the branch found closes an angle that is less then π/4 with the original branch, the two dangling ends are connected with a straight line. If such a connection is not possible, and if it is shorter than 4.2 μm the dangling branch is removed.

Unconnected branches longer than 4.2 μm are extended in the straight line until a connection with the rest of the network is made, if they are found to cut through a middle of a cell that has an area 140% of the average cell in the image. Using this procedure, most of the network becomes enclosed, which completes the mask (Figure 2F).

##### Thresholding

Staining the DNA of the nuclei with Hoechst can result in large intensity variations from one nucleus to another (DNA content, DNA compaction). As a result, a simple threshold applied to the image underestimates the size of darker nuclei and overestimates bright nuclei. To avoid this inconsistency, a local threshold procedure is applied in a three-step fashion. In the first step, a mask is superimposed to the original image, which provides a set of well separated nuclei. In a second step a threshold with a very high value is applied prior to an object search (Figure 2I). Pixels belonging to each nucleus are memorized. In the third step, the mean intensity value of the original image at those pixel positions is calculated. The local threshold value is set to 60% of the original mean intensity value for each nucleus individually (Figure 2J). The objects obtained are used to find the nuclei boundaries (Figure 2K) with an inbuilt MATLAB procedure.

##### Estimation of errors introduced by the segmentation routine

Segmentation issues most often occur at high densities, when two neighboring nuclei are of very different brightness, which results in merging two nuclei into one (undersegmentation), or due to the inhomogeneous intensity of a nucleus, leading to the recognition of two objects (oversegmentation). To evaluate the accuracy of our approach, these errors are corrected manually in 0.65% of cases on a sample of 10,000 cells. Further details, together with the evaluation of segmentation errors for tissues grown on glass and very soft substrates, are shown in the Section 1 of the Supplementary Materials. Importantly, even on very soft substrates (0.6 kPa), where the density is as high as 13,000 cells/mm^2^ our approach recovers 99% of cells correctly, outperforming watershed based algorithms implemented in ImageJ and CellProfiler software packages (Supplementary Section 1).

##### Morphological properties of cell nuclei

The described protocol allows for the accurate determination of a number of morphological measures for each shape (nucleus) in the image including the area and the perimeter. Moreover, centers of mass and orientations of cell nuclei were calculated from binary images using a built-in MATLAB function ‘*regionprops’* contained in the Image Processing Toolbox. This function returns measurements of shape properties for each connected component (nuclei) in the binary image. The connected components are labeled using the flood field algorithm with the connectivity four implemented in the “*bwconncomp”* function.

#### Membrane segmentation

Immuno-fluorescent staining, imaging and segmenting the membrane is a key step for building a reference set of cell shapes used as the source of the “true,” or the so-called “directly measured,” data. Here we use β-catenin staining that reveals the position of the cell membranes and cell-cell contacts. This picture is subdivided into 95 parts and for each part image contrast is increased (intensity histogram in each segment is linearly stretched such that 1% of data is saturated at low and high intensities). At this stage, *h*-minima transform is applied—all intensity minima with an intensity depth that is smaller than the critical value are suppressed using the “imhmin” function in MATLAB. Subsequently, watersheding is performed with pixel connectivity eight using a built-in MATLAB function based on the Fernand-Meyer algorithm.

**Figure 3:**
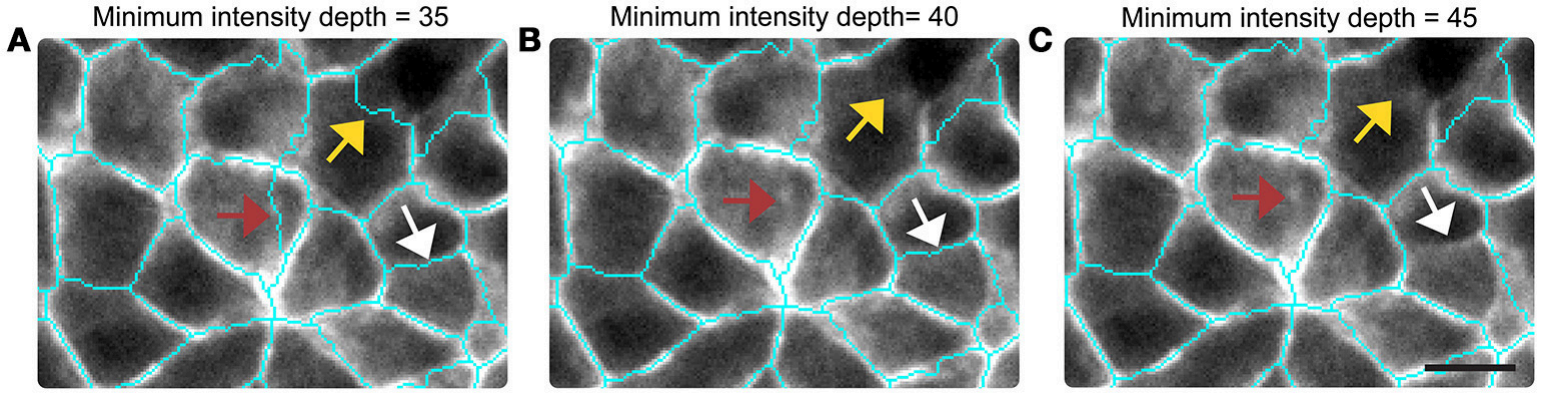
**Sensitivity of the membrane segmentation protocol to the parameters of the watershed algorithm**. Results for several choices of minimum intensity depths are presented **(A–C)**. Arrows point to oversegmented (red) or undersegmented (white, yellow) boundaries. Scale-bar represents 10 μm.

To check for the sensitivity of the segmentation protocol, the analysis is repeated with several critical values for the minima depth (Figures 3A–C, and Supplementary Sections 2, 3). Setting the critical depth of the minima to 35 induced more oversegmentation errors, while the value 45 was associated with significant undersegmentations. Setting the depth to 40 resulted in the correct reconstruction of 98.39% cells. This was determined on a sample of 17,850 cells grown on hard gels by comparison with images that were manually corrected by combining nuclei and membrane pictures. A similar extent of errors is obtained for tissues grown on very soft gels, while larger deviations are generated on glass due to the relatively low intensity of β-catenin on cell-cell contacts at the observed densities (Supplementary Section 2).

Morphological features of cells are obtained from segmented images by a self-developed MATLAB routine. First, vertex pixels of cells are found in the image and sorted in a clockwise direction for each cell. Then all pixels between vertices are detected as boundary pixels. To obtain the boundary length between two vertices, the distance between successive boundary pixels, adopting values of 1 or $\sqrt{2}$, is determined and counted. The perimeter of the cell is the total boundary length between all vertices of the cell. The area is the sum of the pixel areas associated with the object, half of the area associated with boundary pixels and a third of the vertex pixels' area. The number of neighbors is the number of vertices belonging to each cell. Elongation and orientation are obtained with the MATLAB “*regionprops”* function (see above).

### Properties of centre of mass voronoi tessellation

#### Construction of the voronoi tessellation

With a set of distinct points in a continuous space, the Voronoi cell is defined as the region that contains all locations closer to the specific discrete point than to any other (Voronoi, 1908). Even though similar regions were published by Descartes and later by Dirichlet (2D and 3D case), the term Voronoi region is nowadays most commonly used. It is termed after Voronoi who studied those domains in a general *n*-dimensional space. In other words, if *n* centers of mass of nuclei are given:*c*_1_, *c*_2_, …, *c*_*n*_, the Voronoi region associated to the center of mass of the cell nucleus *i* is given by:

$$VT_{i}=\left\{x\in X|d\left(x,c_{i}\right)<d\left(x,\;c_{j}\right)\text{​},\;i\neq j\right\},$$

where *X* is a metric space with a distance function *d* in 2D Euclidian space. An algorithm to compute such tessellations is available online (Barber et al., 1996) and is implemented in C^++^, Python, and MATLAB. We use the software package Computational Geometry for MATLAB based on Qhull for computation of Voronoi tessellations. The set of input parameters are the coordinates of seeding points and output is a list of vertices defining the tessellation. As seeding points we use the centers of mass of all cell nuclei that are completely within the field of view. As a result, one generates a set of polygonal non-intercepting objects, which is intrinsic to CMVT.

#### CMVT morphological measures

For an arbitrarily shaped polygonal object, all morphological measures can be obtained from the positions of the vertices (Goldstein et al., 2002) (Mulchone and Choudhury, 2014). Specifically, for the polygonal cell given by *n* vertices (*x*_*i*_, *y*_*i*_) characteristic for CMVT, the area *A* is given by:

$$A=\frac{1}{2}\sum_{i\;=\;1}^{n}\left(x_{i}y_{i\;+\;1}\;-\;x_{i\;+\;1}\;y_{i}\right),$$

while the perimeter is simply

$$L=\sum_{i=1}^{n}\sqrt{{\left(x_{i}-x_{i+1}\right)}^{2}+{\left(y_{i}-y_{i+1}\right)}^{2}}.$$

The sum runs over a closed path spanned by all vertices and the *n*+1 element in the sum corresponds to first vertex.

The number of neighbors is in principle equal to the number of vertexes, since corrections for vertices shared by more than three cells are negligible in our sample.

The elongation *e* of the cell is calculated from the principle (orthogonal) moments of inertia *I*_1_ and *I*_2_:

$$e=\sqrt{\frac{I_{1}}{I_{2}}}.$$

The moments are obtained from the diagonalization of the inertial tensor with components *I*_*xx*_, *I*_*yy*_, and *I*_*xy*_ calculated in an arbitrary rectangular coordinate system spanning the *xy* plane. As a result one finds:

$$I_{1,2}=\frac{1}{2}(I_{xx}+I_{yy})\pm \sqrt{{\left(I_{xx}+I_{yy}\right)}^{2}\;-\;4(I_{xx}I_{yy}\;-\;I_{xy}^{2})},$$

where *I*_*xx*_, *I*_*yy*_, and *I*_*xy*_ are given by the raw moments of the density distribution within the cell, which is assumed uniform. More specifically:

$$I_{xx}\;=\;m_{02}\;-\;\frac{m_{01}^{2}}{m_{00}},\;I_{yy}\;=\;m_{20}\;-\;\frac{m_{10}^{2}}{m_{00}},\text{ and }I_{xy}\;=\;m_{11}\;-\;\frac{m_{10}m_{01}}{m_{00}}.$$

Zeroth, first and second moments of a regular 2D polygon are listed here:

$$\begin{array}{l} m_{00}=A,\\ m_{10}=\frac{1}{6}\sum_{i=1}^{n}\left(x_{i}+x_{i+1}\right)\left(x_{i}y_{i+1}\;-\;x_{i+1}y_{i}\right),\\ m_{01}=\frac{1}{6}\sum_{i=1}^{n}\left(y_{i}+y_{i+1}\right)\left(x_{i}y_{i+1}\;-\;x_{i+1}y_{i}\right),\\ m_{11}=\frac{1}{24}\sum_{i=1}^{n}\left(x_{i}\;-\;x_{i+1}\right)[x_{i}\left(3y_{i}^{2}\;+\;2y_{i}y_{i+1}\;+\;y_{i+1}^{2}\right)\\ \;+\;x_{i+1}(y_{i}^{2}\;+\;2y_{i}y_{i+1}\;+\;3y_{i+1}^{2})],\\ m_{20}=-\frac{1}{12}\sum_{i=1}^{n}(x_{i}^{3}+x_{i}^{2}x_{i+1}+x_{i}x_{i+1}^{2}+x_{i+1}^{3})(y_{i}\;-\;y_{i+1}),\\ m_{02}=\frac{1}{12}\sum_{i=1}^{n}(y_{i}^{3}+y_{i}^{2}y_{i+1}+y_{i}y_{i+1}^{2}+y_{i+1}^{3})\left(x_{i}\;-\;x_{i+1}\right). \end{array}$$

The standard deviation of from the mean boundary length is given by:

$$\langle △L\rangle =\sqrt{\frac{1}{n}\sum_{i=1}^{n}{\left(\sqrt{{\left(x_{i}\;-\;x_{i+1}\right)}^{2}+{\left(y_{i}\;-\;y_{i+1}\right)}^{2}}\;-\;\frac{L}{n}\right)}^{2}.}$$

The orientation is determined by finding the coordinate system where the off diagonal terms *I*_*xy*_ vanish:

$$\begin{array}{l} I_{{\text{x}}^{\prime }{\text{y}}^{\prime }}=\text{sin}\theta \text{cos}\theta \left(I_{\text{y}}\;-\;I_{\text{x}}\right)+\cos\left(2\theta \right)I_{\text{xy}}=\frac{1}{2}\sin\left(2\theta \right)\left(I_{\text{y}}\;-\;I_{\text{x}}\right)\\ \;+\;\cos\left(2\theta \right)I_{\text{xy}}=0. \end{array}$$

This yields the orientation angle θ

$$\theta =\frac{1}{2}{\text{tan}}^{-1}\left(\frac{-2I_{xy}}{I_{yy}\;-\;I_{xx}}\right).$$

## Results and discussion

### Generation of the sample

The key step in the comparison of CMVT with the true morphology of the cells is the construction of the sample of cells, which will be used for this analysis. Our main sample (hard PA gels) consists of 23 images of cell nuclei and membranes (Supplementary Figures 1, 2). Those images are segmented with the procedure described above providing centers of nuceli (Supplementary Figure 3) as well as outlines of the cell membrane (Supplementary Figure 4). The first criterion that a cell has to satisfy to be part of the set is that it has to have a correctly segmented nucleus as well as a membrane. Yet segmentation disparities are obviously small and amount to about 2% on hard and very soft gels, and about 7% on glass. This comprises the direct error of the nuclei and membrane segmentation (Supplementary Sections 1, 2 for details at different conditions).

Besides correct segmentation, in order to be part of the set, the entire neighborhood of the cell has to be within the field of view. This immediately excludes from the statistics all cells that are at the outer edges of the images (15% on hard gels, 17% on glass, and 11% on very soft gels), since for them, it is not possible to unambiguously reconstruct the tessellation. Moreover, due differences in positions of the cells' nuclei and cell membranes relative to the boundary of the image, there is a 1% difference in the number of cells excluded in the nuclei and membrane channel.

**Figure 4:**
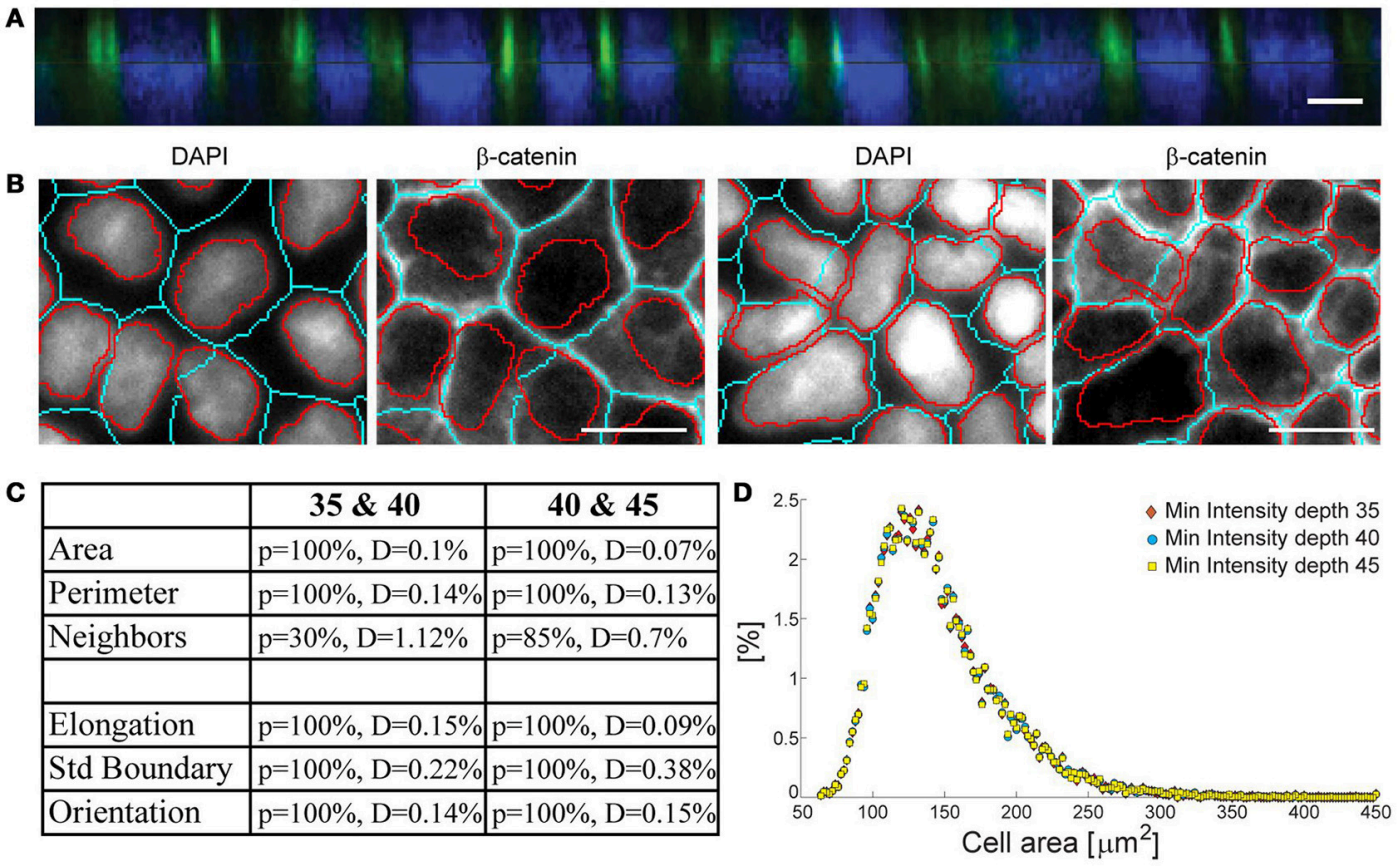
**(A)** Confocal reconstruction (z-x) of β-catenin and the nucleus shows that β-catenin is slightly above the equator of the nucleus. **(B)** Errors intrinsic to the data due to deviations from the epithelial tubular structure, which in 2D projections appear as nuclei protruding into neighboring cells. Scale-bars represent 10 μm. **(C)** Table of K-S test results and **(D)** the distribution of cell areas building the data set, obtained for three different segmentation parameters.

To eliminate false recognitions we, furthermore, introduce the criteria that 95% of the segmented nucleus must be contained inside the segmented membrane, which is not occupied by another nucleus by more than 5% of the total nuclei area. Cells that do not satisfy this criterion do not contribute to the statistics (about 11% for hard PA gels, 28% for soft gels, and 14% for glass). This criterion is introduced to account for the fact that imaging of the membrane and the nuclei require focusing in different planes above the substrate. Namely, adherent junctions associated with β-catenin are, in our samples, positioned slightly above the midline of the cell nucleus (Figure 4A). Therefore, the brightest point of the β-catenin picture can be above the equator of the nucleus along the *z*-axis. If even small deviation from the tubular shape of the cell takes place, the nucleus will appear outside its membrane in the 2D *x-y* projection (Figure 4B). Beside this problem, which is intrinsic to the acquired data, similar effects arise if the threshold value set during the segmentation of a nuclei was set too low. However, this type of error is significantly reduced by the variable threshold introduced in the image analysis.

In the selection procedure described, a total of 16% of cells grown on hard gels, 25% on glass, and 33% on very soft gels are excluded from the statistics. While this is a significant fraction, the advantage of this stringent set of criteria is the insensitivity of the representative set generated on the free parameters in the sampling protocol (Supplementary Section 2.3). This is evident from the assessment of probability distributions calculated for all morphological measures for the three segmentations used in Figure 3. Here, each ensemble of segmented cells is independently subject to the elimination procedure described above, resulting in three representative sets (Figure 4D). These three “true” sets are compared with Kolmogorov-Smirnov (K-S) test providing a *p*-value (probability that two distributions are the same) as well as the maximal distance between two cumulative distribution functions as presented in the table (Figure 4C).

$$D=sup\left|CDF{\left(M^{Memb}\right)}_{1}\;-\;CDF{\left(M^{Memb}\right)}_{2}\right|$$

For example, the distribution of cell areas (Figure 4D) is fully accepted by the K-S test with *p*-value equal to 100%, and the maximal distance between two distributions being 0.32 and 0.2% respectively, showing that all three sets are statistically nearly identical. The number of neighbors, even though it is accepted by the test, has the smallest *p*-value due to discretized nature of this measure. Moreover, this measure is most sensitive to the segmentation errors occurring in the immediate neighborhood of the cell of interest. Most importantly, this analysis shows that the uncertainties of the measured morphological features are very small, and hence can be taken as excellent representatives of the true cell shape characteristics.

**Figure 5:**
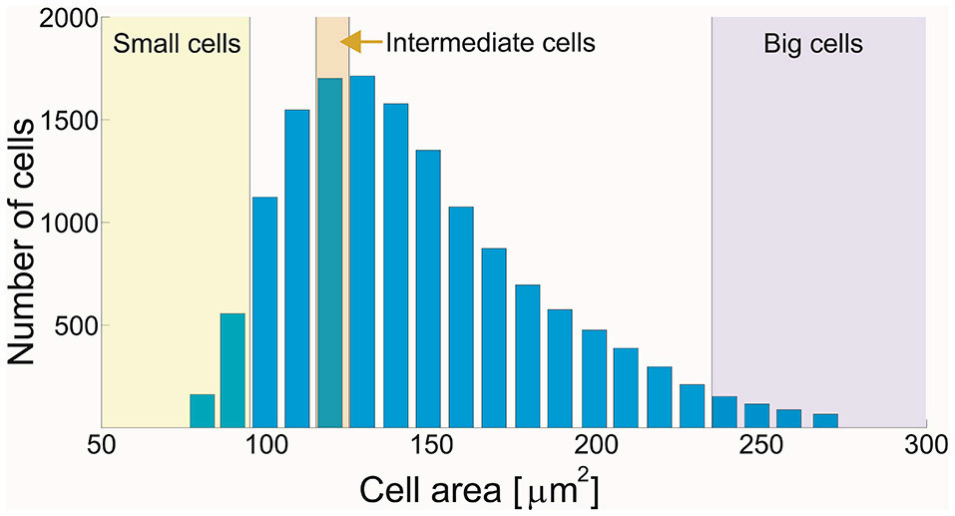
**Distribution of cells in the sample following their area, classified in 20 subsets**.

Our final set consists of 15,014 cells grown on hard substrates, which allows us to study the morphology of cells ranging in area from 74 to 274 μm^2^ with an appropriate statistical accuracy. These cells are classified in 20 subsets according to their size (Figure 5), where each subset has a width of 10 μm^2^ and contains at least 68 cells. For each cell, we determine selected morphological characteristics, first from the images of the membrane and then from CMVT. These findings are then analyzed in detail as described below. Additionally, two smaller sets (covering a smaller range of sizes and/or having smaller statistics) are constructed for testing CMVT on tissues cultivated on glass and very soft gels (Supplementary Sections 4, 5).

### Comparison of morphological measures obtained from membrane images and CMVT estimates

**Figure 6:**
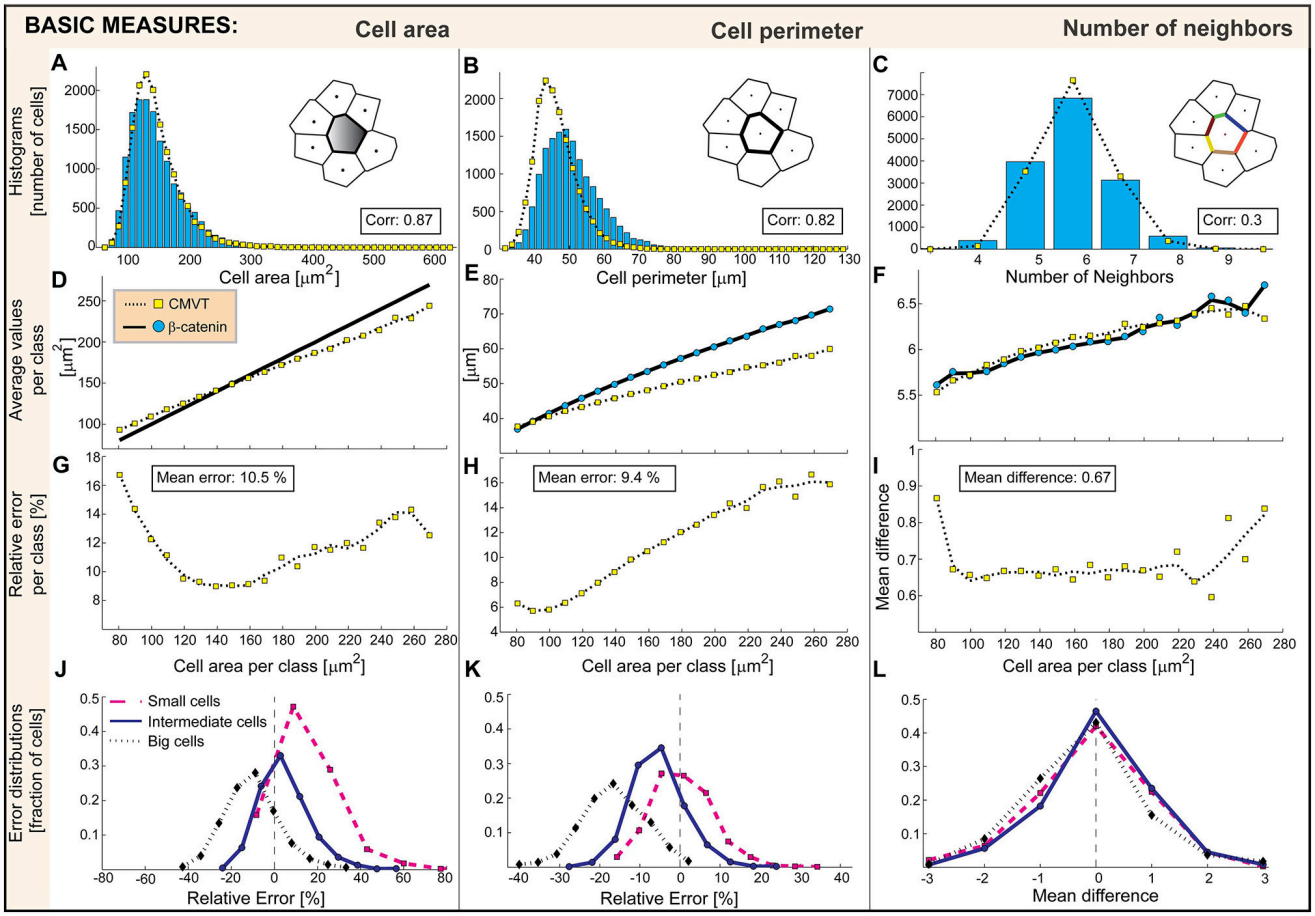
**Comparison of CMVT (yellow squares) and directly extracted morphological measures (blue circles)**. The graphs associated with cell areas, perimeters and number of neighbors are shown in the first, second, and third column, respectively. Top graphs **(A–C)** shows the probability distribution generated by direct measurement and estimated from the tessellation. **(D–F)** Second row is the average dependence of morphological characteristics on the cell area. The associated deviations of CMVT are shown in the third row **(G–I)**. The distributions of errors for small, intermediate-sized and large cells are shown in the bottom row **(J–L)**.

**Figure 7:**
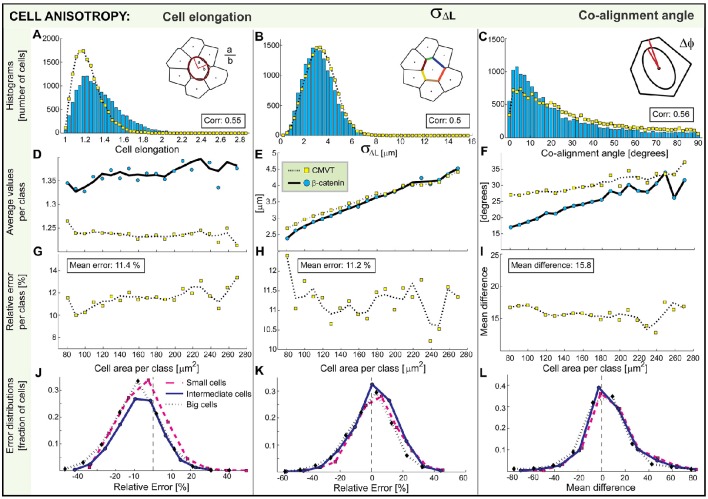
**Comparison of CMVT (yellow squares) and directly extracted morphological measures (blue circles)**. The graphs associated with cell elongation, standard deviation of boundary length and co-alignment of the nuclei and the cell are shown in the first, second, and third column, respectively. Top graphs **(A–C)** shows the probability distribution generated by direct measurement and estimated from the tessellation. **(D–F)** Second row is the average dependence of morphological characteristics on the cell area. The associated deviations of CMVT are shown in the third row **(G–I)**. The distributions of errors for small, intermediate-sized and large cells are shown in the bottom row **(J–L)**.

We first compare the probability distributions of measures emerging from tessellations *M*^*CMVT*^ with directly measured ones from images of the membrane *M*^*Memb*^ (Figures 6A–C) for basic measures such as area, perimeter, and the number of neighbors, and (Figures 7A–C) for anisotropy measures such as the elongation, mean deviation of the contact angle and the co-alignment of principle axis of the cell nuclei and the cell body. The correlation

$$Corr_{CMVT}^{Memb}=\frac{\sum_{i=1}\left(M_{i}^{CMVT}-\langle M^{CMVT}\rangle \right)\left(M_{i}^{Memb}-\langle M^{Memb}\rangle \right)}{\sqrt{\sum_{i=1}{\left(M_{i}^{CMVT}-\langle M^{CMVT}\rangle \right)}^{2}}\sqrt{\sum_{i=1}{\left(M_{i}^{Memb}-\langle M^{Memb}\rangle \right)}^{2}}}$$

between the two measured and the CMVT estimated distributions of a morphological measure is shown in the inset. Here the average in the bracket denotes the average of the respective distributions and the sum runs over all cells in the set.

Furthermore, sorting by the measured areas (Figure 5), we build 20 subclasses (indexed with *k*), each containing *N*_*k*_ cells (*N*_*k*_ > 68 and every cell in the subclass is denoted by an index *i* = 1 …*N*_*k*_). The mean value of a particular morphological measure $\langle M_{k}^{CMVT}\rangle$ and $\langle M_{k}^{Memb}\rangle$ is presented as function of the mean cell area in each subclass and shown in the second row of Figures 6D–F, 7D–F.

We compare data on cell-by-cell basis and calculate the mean relative error $ErrM_{k}^{CMVT}$ of a measure *M*^*CMVT*^ comparative to *M*^*Memb*^ in each subclass:

$$ErrM_{k}^{CMVT}=N_{k}^{-1}\sum_{i=1}^{N_{k}}\left|M_{i}^{CMVT}-M_{i}^{Memb}\right|/M_{i}^{Memb}$$

This error measure (third rows—Figures 6G,H, 7G,H) denotes the average deviation of the CMVT estimated from the measured magnitude of a morphological characteristic of interest as a function of the mean cell area in a particular subclass. Exceptionally, for the number of neighbors (Figure 6I), and the co-alignment (Figure 7I) we report the mean difference

$$\langle \Delta {\text{M}}_{\text{k}}^{\text{CMVT}}\rangle \;=\;N_{\text{k}}^{-1}\sum_{\text{i}=1}^{{\text{N}}_{\text{k}}}\left|{\text{M}}_{\text{i}}^{\text{CMVT}}{\text{ - M}}_{\text{i}}^{\text{Memb}}\right|.$$

In the insets of the graphs, we show the mean tessellation error calculated for all of *N* cells in the set

$$\langle ErrM^{CMVT}\rangle =\frac{\frac{1}{N}\sum_{i=1}^{N}\left|M_{i}^{CMVT}-M_{i}^{Memb}\right|}{M_{i}^{Memb}}$$

$$=\sum_{k=1}^{20}\frac{N_{k}}{N}ErrM_{k}^{CMVT}.$$

In the case of number of neighbors and co-alignment of the nuclei and cell body, we calculate the mean difference

$$\langle ErrM^{CMVT}\rangle =\frac{1}{N}\sum_{i=1}^{N}\left|M_{i}^{CMVT}\;-\;M_{i}^{Memb}\right|,$$

which averages the deviation over all cells in the sample.

Finally, we show the distribution of relative errors ($M_{i}^{CMVT}-M_{i}^{Memb}$)/$M_{i}^{Memb}$ (Figures 6J,K, 7J,K) or the distribution of differences (Figures 6L, 7L) in the bottom row for several subsets of cells. Here we focus on particularly small and large cells, as well as a set of cells of intermediate size (as indicated in Figure 5), to see what type of cells actually contribute to the error of the tessellation.

### Basic measures

The most commonly discussed morphological characteristic of cells in a tissue is the average area or cell density. The analysis of CMVT prediction shows that the distribution of cell area is reasonably well reproduced. This agreement is confirmed by the comparison of the probability distributions of the areas measured and areas of cells obtained from the tessellation (Figure 6A), and the relatively high degree of correlation between the two distributions. However, further analysis over the subclasses (Figure 6D), shows that the areas of larger cells are systematically underestimated, and the areas of smaller cells overestimated by the tessellation. Accordingly, the distributions of errors presented for small, midsized, and large cells (Figure 6J) are not centered at zero. For small cells the offset is toward positive values, while for large cells is it toward negative values showing a systematic error of the tessellation that makes small cells larger and large cells smaller. Consequently, the mean subpopulation error increases toward the two extrema in cell sizes (Figure 6G). Nevertheless, the areas of the cells are reasonably well reproduced by CMVT, and the mean error is about 10%. Notably, the mean cell size of the set is estimated with 0.25% error, which justifies the utilization of CMVT in estimations of the mean cell density, a result that should be seen in the light of the uncertainty of the *mean* “true” area 〈*M*^*Memb*^〉 of 0.04% (Figure 4D).

Significantly stronger deviations of CMVT from the true data can be seen in the distribution of perimeters (Figure 6B). This measure is mainly affected by the curved nature of the cell wall, which the tessellation approximates with a straight line. Consequently, the length of the boundaries is systematically underestimated, shifting the whole distribution to smaller values. Naturally, best performance is obtained for relatively small cells (Figure 6E). Nonetheless, the mean error of the tessellation is only 9.4%. Moreover, since the perimeter of the cell is strongly correlated with the cell area, the systematic size dependent errors prevail and are even somewhat enhanced (Figures 7E,H,K). This is particularly acute for large cells where CMVT underestimates the cell perimeter more than 16% on average (Figure 7H).

The distribution of the number of neighbors (Figure 6C) is well accounted for by the tessellation, even though the number of cells having 6 neighbors is systematically overestimated. The distribution is slightly skewed for both measured and tessellated data sets, and the dependence on the cell size (Figure 6F) is well reproduced. The more detailed analysis of the errors generated (Figure 6L) shows that despite centering at zero, overestimates of the number of neighbors are more common for intermediate cell sizes, unlike for very large, and small cells that tend to underestimate the number of neighbors. In fact, on average, in 22.3% of cases CMVT overestimates, and in 20% underestimates the number of neighbors by one. Actually, CMVT correctly estimates the number of neighbors only for 46.5% of cells, which yields a low correlation index. This poor performance is also associated with the segmentation errors in the immediate environment of the cell of interest. Therefore, we conclude that CMVT provides the mean number of neighbors in a quantitative manner, but not on a single cell level.

### Measures of cell anisotropy

Several measures such as the elongation, the standard deviation of boundary length and the co-alignment angle between the principle axis of the cell and its nucleus are all sensitive to the anisotropic properties of the cell shape. For the cell elongation and the co-alignment of the cell's body and nuclei CMVT only qualitatively represents the probability distributions (Figures 7A,C). As in the case of perimeters, these measures show systematic errors. While the mean error of the tessellation remains between 10 and 15%, the correlation between the measured and the estimated distributions remains only about 0.5. Interestingly, the elongation seems to be independent of the cell size and is around 1.3, similar to previous reports (Puliafito et al., 2012). However, it is systematically underestimated by CMVT—the cells turn out more spherical than they are (Figure 7D). Consequently, the distribution of errors is negatively skewed (Figure 7J). At the same time, the co-alignment between the cell and its nuclei is underestimated by the tessellation (Figure 7F). Naturally, the distribution of errors (Figure 7L) is positively skewed, even though the maximum of the distribution is still around zero. Overall, these results suggest that in isotropic cells no particular nuclear polarity takes place, as expected, a result that is captured by CMVT but only on average and not on the single cell level. However, since most cells in this tissue have an elongated shape, associated with the co-alignment of the cell and the nuclei, significant errors are generated by the tessellation. The variation of boundary length, which should be larger for elongated cells then for isotropic cells, itself increases linearly with the cell area (Figure 7E), which is interesting in the light of the insensitivity of the elongation to the cell area, and the fact that linear increase is expected with the cell perimeter. Importantly, CMVT well reproduces this trend, but still has mean error of 11.2% on the level of the single cell.

Unlike basic measures, the morphology measures associated with cell anisotropy do not show errors that are strongly size dependent, but seem systematic. Consequently, the distributions of errors do not change shape for different subclasses of cell sizes (Figures 7J–L).

### Cross-correlations between measures

From the previous discussion, it becomes evident that there must be a degree of correlations between various morphological measures. To quantify this, we calculate the correlation coefficient ${Corr}_{M^{a}}^{M^{b}}$, which measures the extent of the linear relationship between two estimated or directly measured properties of the cells' shape *M* ^*a*^ and *M* ^*b*^:

$$Corr_{M^{a}}^{M^{b}}=\frac{\sum_{i=1}\left(M_{i}^{a}-\langle M^{a}\rangle \right)\left(M_{i}^{b}-\langle M^{b}\rangle \right)}{\sqrt{\sum_{i=1}{\left(M_{i}^{a}-\langle M^{a}\rangle \right)}^{2}}\sqrt{{\sum_{i=1}\left(M_{i}^{b}-\langle M^{b}\rangle \right)}^{2}}}.$$

**Figure 8:**
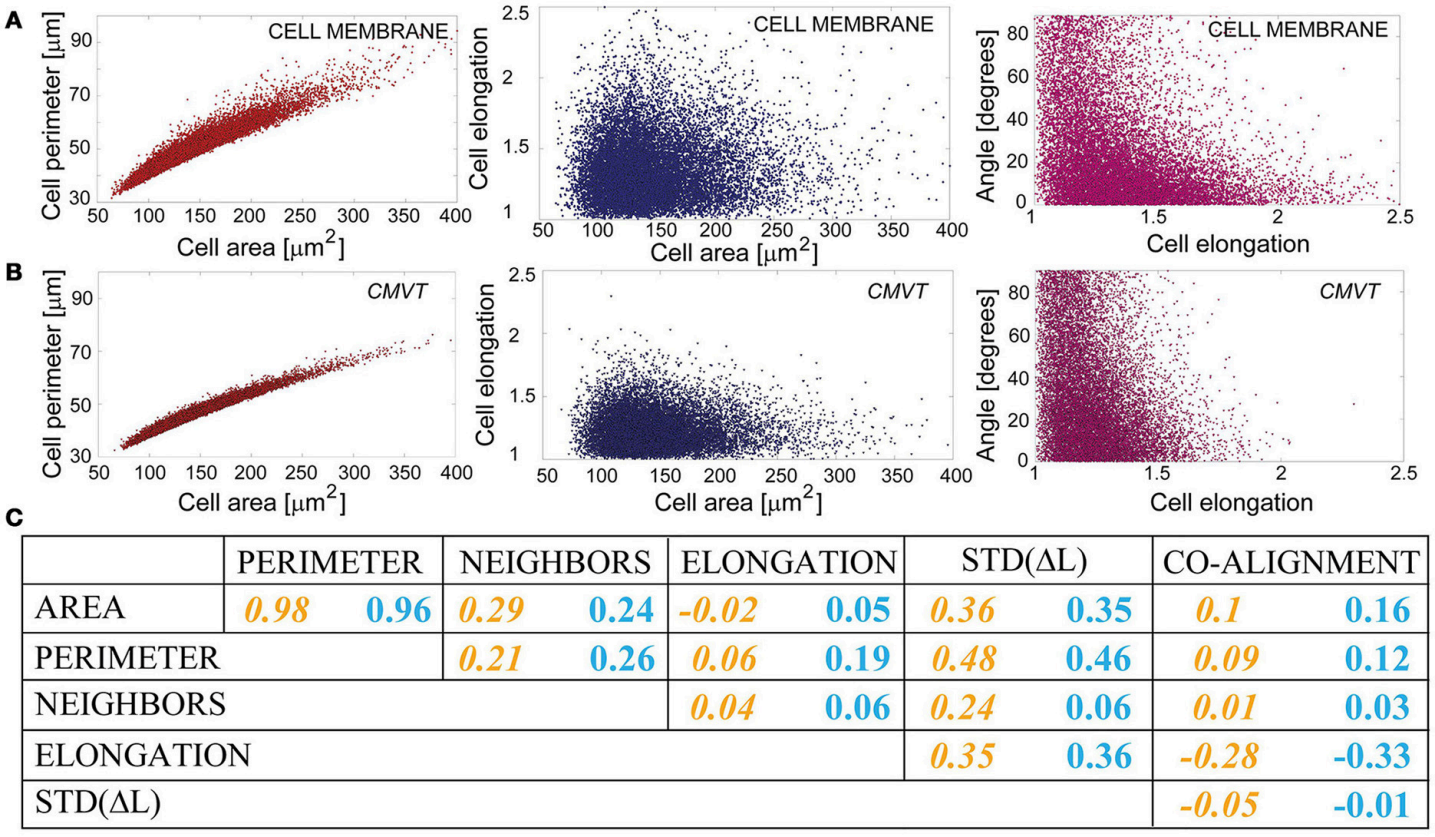
**Scatter plots and cross-corelations**. **(A,B)** Exemplary scatter plots showing the cross-correlation (left panels) or the lack of it (middle and the right panels) between various morphological measures. **(C)** Correlation coefficients between two morphological measures, as estimated by CMVT (yellow numbers on the left) and directly measured (blue numbers on the right in each column). Cross-correlation coefficient can adopt values between −1 for anti-correlated measures to 1 for fully correlated ones.

The mean in the brackets denotes the mean of the distributions shown in Figures 6A–C and Figures 7A–C, and the sum is performed over all cells in the set. The correlation coefficients as defined above are identical to elements of the normalized covariance matrix of the data set. The latter is commonly used as a starting point for the multivariate data analysis, which can provide more detailed insights into the correlations within the statistical set. However, already the analysis of the provided scatter plots and cross correlation coefficients (Figure 8 and Supplementary Section 6) may provide useful information. For example, it is clear that CMVT captures appropriately the level cross-correlation between various measures.

Interestingly, the only strong cross-correlation in the data is between the cells' areas and perimeters, evidenced from scatter plots shown in Figures 8A,B. The cross-correlation is even overestimated by CMVT (Figure 8C), due to the inability of CMVT to capture small fluctuations of the cell boundary arising in the true data.

Surprisingly, cell areas are not strongly cross-correlating with any other measures, neither in the true set, nor in the CMVT reconstruction. Weak cross-correlations exist with the number of neighbors, and the standard deviations in the boundary length. The latter is the consequence of cross-correlation between perimeters and the variations in the boundary lengths, which is most likely of purely geometric origin, as well as the weak cross-correlations between the variation in boundary length and the cell elongation. Interestingly, we find that the co-alignment of the cell nuclei and the cell body does not cross-correlate with the cell area, despite the expectation that in smaller cells, stress on the nuclei will be strongly coupled to the stress on the cell membrane. Likewise, no appreciable relation between the cell area and the cell elongation emerges from our data. This trend is well reproduced by CMVT.

In the context of other anisotropy measures, it was already anticipated in the previous section that elongation of the cells anti-correlates with the co-alignment of the cell and its nuclei. In other words, in elongated cells, the orientation of the nuclei follows the orientation of the cell, whereas in more isotropic cells, this correlation is lost. This trend is well captured by CMVT, as well as the lack of correlation of these two measures with other characteristics of the cell shape.

## Discussion and conclusions

The aim of this work was to establish a method for a systematic comparison of the true morphology of a tissue and the CMVT estimate, which is an important problem in the physiology of epithelium. We first applied this procedure to MDCK cells monolayers grown on relatively hard gels where the tessellation reproduces various shape characteristics with a mean error of 10 to 15% and qualitatively correctly captures the cross-correlations between various measures. We find that the tessellation predicts the mean cell area with very high accuracy, thus validating the common use of CMVT in estimating the macroscopic cell density. However, the cell-by-cell analysis reveals significant cell-size related effects, and relatively large deviations for the sub-populations of small and large cells. Interestingly, the same trends are recovered in tissues grown on glass and on very soft substrates (Supplementary Sections 4, 5). Systematic comparison shows that the smallest deviations in the estimates of area occur close to the peak of the area distributions in all growing conditions, which is the foundation for the CMVT applicability.

Several other observations are independent of growth conditions. For example, significant deviations of CMVT, dependent on cell sizes occur in the determination of the number of neighbors, a fact that can also be associated in part with errors in segmentation. However, for all substrates, CMVT over-stabilizes the honeycomb structures with six neighbors. Furthermore, characteristics reflecting the anisotropy of cells such as the elongation, variation in cell boundary lengths, or the co-alignment of the cell body and the cell nuclei, are only qualitatively reproduced by CMVT in all conditions, albeit correct trends are usually recovered in the context of the dependence on the cell size. The largest discrepancies between CMVT and the true shape characteristics occur for particularly large or small cells. This is evident from the relative errors of perimeters in the subpopulation of the large cells, and relative errors of areas in the subpopulation of the small cells, being more than 16%.

Interestingly, averages of most morphological measures over sub-populations of cells, sorted by size, depend linearly on the associated mean cell areas, suggesting different scaling factors for each measure (Supplementary Section 7). However, such scaling may be applied only to averages generated for particular cell sizes, because the distributions of errors in each sub-population are typically very broad and cross-correlation factors small. The exception are the cross-correlations between cell perimeters and areas. Consequently, correction scaling should not be generally applied on the isolated cell level.

To validate the use of CMVT we developed a procedure that requires building a representative set of cells from which the ‘true’ distributions of morphological measures can be extracted. Interestingly, we find that these distributions converge only for sets comprising over 10,000 cells, as exemplified here by distributions generated for cells grown on hard gels. Sets with only a few thousands of cells (as achieved on glass and soft substrates) are, on the other hand, only able to predict trends and the mean errors, but correlation coefficients between the CMVT predictions and the true distributions are strongly affected by the inaccuracy of the reference set.

Generating such large statistics may be difficult in some cases. For example, monolayers of MDCK cells grown on collagen coated glass contain significantly more defects then on hard gels, affecting the segmentation of the membrane and making the generation of the reference set more difficult. Similarly, on soft substrates, the high density of cells makes the segmentation of nuclei cumbersome, requiring the development of more accurate procedures. Actually, such large number of cells call for the development of fully automated, yet competitive segmentation procedures, an example of which is presented here in the context of extraction of cell nuclei.

The establishment of the current validation method is the prerequisite for further analysis of the morphology of MDCK cells and of other tissues. In particular, with the appropriately constructed reference set, one could use the CMVT to delineate between the well-characterized and unknown tissue (e.g., healthy and cancerous growth), or between different stages in development. The current findings and the limitation of CMVT should also be considered in theoretical modeling of epithelial tissues where these types of tessellations are commonly applied to generate the growing structures.

In the context of MDCK cells, it would be interesting to explore the performance of the CMVT as a function of the more controlled density of cells, also associated with a smaller range of cell sizes. This would provide better understanding of cellular mechanics and spatial correlations within the tissue.

One should however keep in mind that the largest source of errors in CMVT predictions of cell morphologies is due to the polygonal nature of this tessellation. Consequently, after establishing a reference set, the correspondence between the measured and estimated morphological characteristics cannot be significantly improved with more precise imaging. Therefore, even though a reasonable agreement is obtained, more sophisticated tessellation methods, which systematically estimate all shape features, should be tested in future.

## Author contributions

SK constructed and performed the image analysis. FR directed experiments. CJ cultured cells and acquired the images. AS conceived and supervised the project. SK and AS wrote the paper.

## Funding

FR acknowledges funding by the Deutsche Forschungsgemeinschaft (DFG) through SFB 755, project B08. AS received funding from the European Research Council (StG MembranesAct 2013-337283). AS and SK acknowledge the support by the Research Training Group 1962 at the FAU Erlangen–Nürnberg.

### Conflict of interest statement

The authors declare that the research was conducted in the absence of any commercial or financial relationships that could be construed as a potential conflict of interest. The handling Editor declared a shared affiliation, though no other collaboration, with several of the authors SK and AS and states that the process nevertheless met the standards of a fair and objective review.

## References

[B1] Angelini T. E. Hannezo E. Trepat X. Marquez M. Fredberg J. J. Weitz D. A. (2011). Glass-like dynamics of collective cell migration. Proc. Natl. Acad. Sci. U.S.A. 108, 4714–4719. 10.1073/pnas.1010059108 21321233 PMC3064326

[B2] Barber C. B. Dobkin D. P. Huhdanpaa H. T. (1996). The Quickhull algorithm for convex hulls. ACM Trans. Math. Softw. 22, 469–83.

[B3] Dapeng B. Lopez J. H. Schwarz J. M. Manning L. (2015). A density-independent rigidity transition in biological tissues. Nat. Phys. 11, 1074–1079. 10.1038/nphys3471

[B4] Darro F. Kruczynski A. Etievant C. Martinez J. Pasteels J. L. Kiss R. (1993). Characterization of the differentiation of human colorectal cancer cell lines by means of Voronoi diagrams. Cytometry 14, 783–792. 10.1002/cyto.990140711 8243207

[B5] Das T. Safferling K. Rausch S. Grabe N. Boehm H. Spatz J. P. (2015). A molecular mechanotransduction pathway regulates collective migration of epithelial cells. Nat. Cell Biol. 17, 276–287. 10.1038/ncb3115 25706233

[B6] Deforet M. Hakim V. Yevick H. G. Duclos G. Silberzan P. (2014). Emergence of collective modes and tri-dimensional structures from epithelial confinement. Nat. Commun. 5, 3747. 10.1038/ncomms4747 24796352

[B7] Farhadifar R. Röper J. C. Aigouy B. Eaton S. Jülicher F. (2007). The influence of cell mechanics, cell-cell interactions, and proliferation on epithelial packing. Curr. Biol. 17, 2095–2104. 10.1016/j.cub.2007.11.049 18082406

[B8] Goldstein H. Poole C. P. Safko J. (2002). The rigid body equations of motion in Clasical Mechanics (New Delhi: Addison-wesley), 184–238.

[B9] Guillaud M. Buys T. P. Carraro A. Korbelik J. Follen M. Scheurer M.. (2014). Evaluation of HPV infection and smoking status impacts on cell proliferation in epithelial layers of cervical neoplasia. PLoS ONE 9:e107088. 10.1371/journal.pone.0107088 25210770 PMC4161429

[B10] Hannezo E. Prost J. Joanny J. F. (2014). Theory of epithelial sheet morphology in three dimensions. Proc Natl. Acad. Sci. U.S.A. 111, 27–32. 10.1073/pnas.1312076111 24367079 PMC3890844

[B11] Harris A. R. Petera L. Bellise J. Baume B. Kablag A. J. Charrasa G. T. (2013). Characterizing the mechanics of cultured cell monolayers. Nat. Protoc. 8, 2516–2530. 10.1073/pnas.1213301109 24263091

[B12] Honda H. (1978). Description of cellular patterns by Dirichlet domains: the two-dimensional case. J. Theor. Biol. 72, 523–543. 10.1016/0022-5193(78)90315-6 672241

[B13] Kaliman S. Jayachandran C. Rehfeldt F. Smith A. S. (2014). Novel growth regime of MDCK II model tissues on soft substrates. Biophys. J. 106, L25–L28. 10.1016/j.bpj.2013.12.056 24703316 PMC3976519

[B14] Kayser K. Stute H. (1989). Minimum spanning tree, Voronoi's tesselation and Johnson-Mehl diagrams in human lung carcinoma. Pathol. Res. Pract. 185, 729–734. 10.1016/S0344-0338(89)80228-6 2560544

[B15] Malpica N. de Solórzano C. O. Vaquero J. J. Santos A. Vallcorba I. Gracia-Sagredo J. M.. (1997). Applying watershed algorithms to the segmentation of clustered nuclei. Cytometry 28, 289–297. 10.1002/(SICI)1097-0320(19970801)28:4<289::AID-CYTO3>3.0.CO;2-7 9266748

[B16] Meijering E. (2012). Cell segmentation: 50 years down the road. IEEE Signal. Proc. Mag. 29, 140–145. 10.1109/MSP.2012.2204190

[B17] Mkrtchyan A. Åström J. Karttunen M. (2014). A new model for cell division and migration with spontaneous topology changes. Soft Matter 10, 4332–4339. 10.1039/c4sm00489b 24793724

[B18] Mulchone K. F. Choudhury K. R. (2014). Fitting an ellipse to an arbitrary shape: implications for strain analysis. J. Struct. Geol. 26, 143–153. 10.1016/S0191-8141(03)00093-2

[B19] Ntziachristos V. (2010). Going deeper than microscopy: the optical imaging frontier in biology. Nat. Methods 7, 603–614. 10.1038/nmeth.1483 20676081

[B20] Puliafito A. Hufnagela L. Neveua P. Streichanb S. Sigalc A. Fygensond D. K.. (2012). Collective and single cell behavior in epithelial contact inhibition. Proc Natl. Acad. Sci. U.S.A. 109, 739–744 10.1073/pnas.1007809109 22228306 PMC3271933

[B21] Sheikhzadeh F. Ward R. K. Carraro A. Chen Z. Y. van Niekerk D. Miller D.. (2015). Quantification of confocal fluorescence microscopy for the detection of cervical intraepithelial neoplasia. Biomed. Eng. Online. 14, 96. 10.1186/s12938-015-0093-6 26499452 PMC4619300

[B22] Streichan S. J. Hoerner C. R. Schneidt T. Holzer D. Hufnagel L. (2014). Spatial constraints control cell proliferation in tissues. Proc Natl. Acad. Sci. U.S.A. 111, 5586–5591. 10.1073/pnas.1323016111 24706777 PMC3992650

[B23] Sudbø J. Marcelpoil R. Reith A. (2000). New algorithms based on the Voronoi Diagram applied in a pilot study on normal mucosa and carcinomas. Anal. Cell. Pathol. 21, 71–86. 10.1155/2000/389361 11310643 PMC4618427

[B24] Sulsky D. Childress S. Percus J. K. (1984). A model of cell sorting. J. Theor. Biol. 106, 275–301. 10.1016/0022-5193(84)90031-6 6717032

[B25] Trepat X. Wasserman M. R. Angelini T. E. Millet E. Weitz D. A. (2009). Physical forces during collective cell migration. Nat. Phys. 5, 426–430. 10.1038/nphys1269

[B26] Vincent L. Soille P. (1991). Watersheds in digital spaces: an efficient algorithm based on immersion simulations. IEEE T Pattern Anal. 13, 583–588. 10.1109/34.87344

[B27] Voronoi G. (1908). Nouvelles applications des paramètres continus à la théorie des formes quadratiques. J. Reine Angew. Math. 133, 97–178. 10.1515/crll.1908.133.97

[B28] Wienert S. Heim D. Saeger K. Stenzinger A. Beil M. Hufnagl P.. (2012). Detection and segmentation of cell nuclei in virtual microscopy images: a minimum-model approach. Sci. Rep. 2:503. 10.1038/srep00503 22787560 PMC3394088

[B29] Xing F. Yang L. (2016). Robust nucleus/cell detection and segmentation in digital pathology and microscopy images: a comprehensive review. IEEE Rev. Biomed. Eng. 9, 234–263. 10.1109/RBME.2016.2515127 26742143 PMC5233461

[B30] Zehnder S. M. Suaris M. Bellaire M. M. Angelini T.E (2015). Cell volume fluctuations in MDCK monolayers. Biophys. J. 108, 247–250. 10.1016/j.bpj.2014.11.1856 25606673 PMC4302189

[B31] Zhang C. Sun C. Pham T. D. (2013). Segmentation of clustered nuclei based on concave curve expansion. J. Microsc. 251, 57–67. 10.1111/jmi.12043 23692597

[B32] Zorn M. L. Marel A. K. Segerer F. J. Rädler J. O. (2015). Phenomenological approaches to collective behavior in epithelial cell migration. Biochim. Biophys. Acta 1853, 3143–3152. 10.1016/j.bbamcr.2015.05.021 26028592

