# Supplementary material for: Limits of Applicability of the Voronoi Tessellation Determined by Centers of Cell Nuclei to Epithelium Morphology
Source: Front Physiol. 2016 Nov 25;7:551. doi: 10.3389/fphys.2016.00551 (PMC5122581; doi:10.3389/fphys.2016.00551)
Supplement: Supplementary file 1 [file Presentation1.PDF]

Supplementary Material

Limits of applicability of the Voronoi tessellation determined by centers of cell nuclei to epithelium morphology

Sara Kaliman [1], Christina Jayachandran [2], Florian Rehfeldt [2], Ana-Sunčana Smith [1,3*]

*** Correspondence:** Corresponding Author: smith@physik.fau.de

Table of content:

1. Nuclei segmentation routine

1.1. Cells on hard PA gels

1.2. Cells on soft PA gels

1.3. Cells on glass substrate

2. Error of the membrane segmentation

2.1. Counting segmentation errors

2.2. Consistency of the error estimations

2.3. Choosing the correct value of the suppressed minima in h-minima transform

3. Segmentation procedure for MDCK II monolayer grown on hard PA gels

4. Performance of CMVT on soft PA gels

5. Performance of CMVT on glass substrate

6. Scatter plots

7. Linear regression of the mean values

1 Nuclei segmentation routine

We compare our segmentation procedure to ImageJ and CellProfiler, which are the two commonly used open-source software toolboxes for image analysis of cells:

ImageJ officially recommends a specific procedure for the segmentation of DAPI stained nuclei[1]. Accordingly, first a Gausian blur filter should be used, before generating a binary image. Then, watershed is applied to a distance-transformed binary image. Since the Gaussian blur filter did not yield satisfactory results, we changed this approach to first subtracting the background (rolling ball radius of 60 pixels). The resulting image is binarized and watershed is applied as suggested by the developers.

CellProfiler offers a segmentation tool that can be self-tailored to the properties of the objects expected in the image. This segmentation procedure includes methods for distinguishing clumped objects (segment objects) and methods for drawing lines between clumped objects (properly separate segmented objects)[2]. We obtain the best segmentation results with the so called ‘Intensity’ method in both cases. In the case of distinguishing clumped objects, the ‘Intensity’ algorithm counts each intensity peak as a separate object and is particularly suitable for objects that are brighter towards their interiors. For drawing lines between clumped objects, the ‘Intensity’ method performs a watershed on the original image starting from previously identified local maxima as seeds. Final thresholding of the image is performed using the Otsu method.

The performance of these two segmentation approaches is compared to the self-developed procedure described in the manuscript in the section 2.3.1 on images of Hoechst stained images (325.3 x 434.2 μm, pixel size 0.31 μm) of nuclei. The comparison is performed on images of cells grown on hard polyacrylamide (PA) gels (E > 3kPa), soft PA gels (E = 0.6 ± 0.2 kPa), and on glass. Over-segmentation and under-segmentation errors (Supplementary Figure 1) are counted manually.

1.1 Cells on hard PA gels

The tested set of data consists of ten images with various cell densities. The set is drawn from the original set of twenty-three images. Approximately 10,000 cells are analyzed with our method, ImageJ, and Cell Profiler. Oversegmentation and undersegmentation errors (Supplementary Figure 1) were manually counted. Results show that our method is prone to less errors than ImageJ and CellProfiler (Supplementary table 1) with a total error of 0.65%. The two watershed based algorithms had errors of 1.53% and 2.28%. ImageJ is significantly more prone to oversegmentation errors, which results in overestimation of an overall cell density. CellProfiler on the other hand is much slower than our method and ImageJ and tends to undersegment cells.

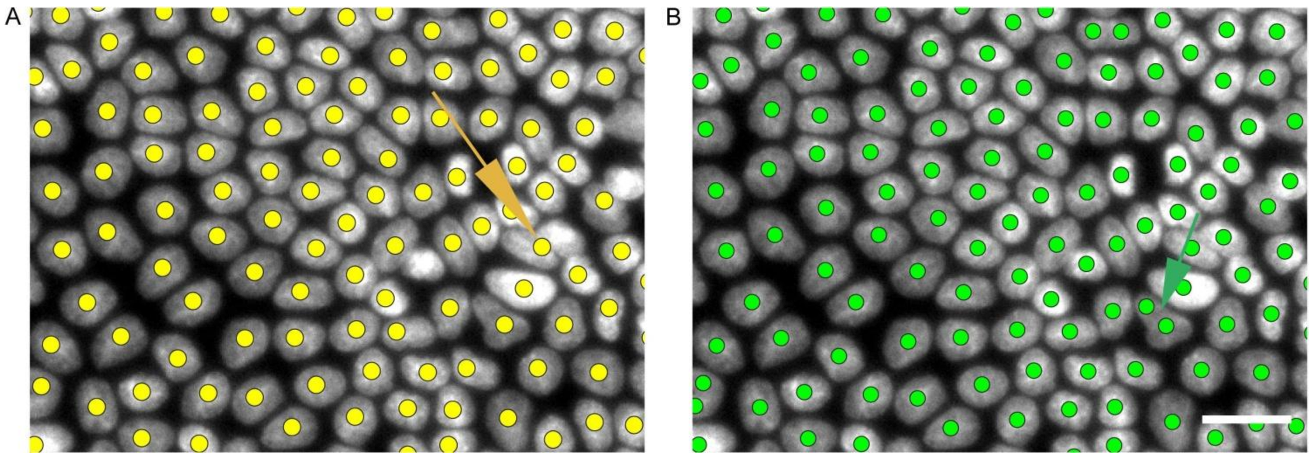

Supplementary Figure 1. A) Typical undersegmentation error. B) Typical oversegmentation error. Scale bar is 20 μm.

Table 1. Nuclei segmentation errors on hard PA gels.

**hard PA gels (10,386 cells)**	Undersegmentation	Oversegmentation	Total error
Our method	0.32	0.34	0.65
CellProfiler	1.41	0.12	1.53
ImageJ	0.4	1.88	2.28

1.2 Cells on soft PA gels

We analyzed three images used in the analysis (Supplementary information 3). Those images contained about 5,000 cells. Since the cells and their respective nuclei are very densely packed on the soft PA gels ($E = 0.6 \pm 0.2$ kPa), all three methods have the largest errors for these conditions. However, our segmentation method was superior to ImageJ and CellProfiler (Supplementary table 2) with a total error of 1.02% in comparison to 3.54% and 2.51%. CellProfiler tended to do more under segmentation errors same as in case of the main data set. ImageJ, on high densities, is equally prone to under and over segmentation of the cells' nuclei.

Table 2. Nuclei segmentation errors on soft PA gels.

**soft PA gel (5,216 cells)**	Undersegmentation	Oversegmentation	Total error
our method	0.9	0.12	1.02
CellProfiler	3.37	0.17	3.54
ImageJ	1.21	1.3	2.51

1.3 Cells on glass substrate

Six images used for analysis (Supplementary information 4) were tested for segmentation errors. In total, approx. 5,000 cells are analyzed. Those images had the smallest cell density of 5,200 ± 400 cells/mm^2, and therefore all three methods performed the best on those images. Our method was again better than CellProfiler and ImageJ (Supplementary table 3). CellProfiler tends to over segment images of cells grown on glass, in contrast to results on hard PA gels. ImageJ has the same tendency to over and under segment images as on soft Pa gels. Our method, on the other hand, has a tendency to over and under segment cell nuclei on all substrates leading to very small error in density estimation on all images.

Table 3. Nuclei segmentation errors on glass substrates.

**glass (3,965 cells)**	Undersegmentation	Oversegmentation	Total error
Our method	0.05	0.08	0.13
CellProfiler	0.07	0.28	0.35
ImageJ	0.13	0.1	0.23

2 Error of the membrane segmentation

2.1 Counting segmentation errors

Same as in the case of the nuclei segmentation, we have counted manually the number of under and over segmentation cases in the segmentation of our β-catenin images. The counting is done on all images used in the analysis: twenty-three in the case of hard PA gels, three on soft Pa gels, and six images of the cells grown on glass substrate. Data is presented in the Supplementary table 4. We see that for our main set of data, the segmentation error was 1.61%. On soft PA gels, segmentation is much more precise (0.73% of errors), while on glass substrates segmentation of β-catenin images leads very often to over segmentation errors (6.01% of over segmentation errors).

Table 4. Membrane segmentation errors on all substrates.

**substrate**	Undersegmentation	Oversegmentation	Total error
soft PA gels	0.42	0.31	0.73
hard PA gels	0.68	0.93	1.61
glass	0.91	6.1	7.01

2.2 Consistency of the error estimations

We compare consistency of manually counted estimates of the segmentation errors with automatic counting of the cells removed from the statistics because of our criteria (see section 3.1 in the manuscript), and find that numbers match. In the case of our main data set (hard PA gels), from total number of cells removed from the statistic, 70% had one to one correspondence between membrane and cell's nucleus, but they did not satisfy the criteria that 95% of the nucleus is in the membrane and the membrane is not occupied by other nucleus by more than 5% of the nucleus area. The remaining 30% of membranes removed from the statistic did not have one associated cell nucleus (20% had no nucleus inside and 10% had more than 1). Since in total 15.6% of membranes are eliminated, we see that 4.7% of total number of membranes are eliminated because one to one correspondence could not be established. From that number, 1% of the membranes are eliminated due to the difference in the statistics of the segmented membranes and nuclei, which arises from slightly different elimination criteria of the cells at the edge. The rest of the 3.7% is consistent with our table of manually counted errors in over and under segmentation of cells' nuclei and cells' membranes. The total number of errors is 3.3% (nuclei over segmentation and membrane under segmentation need to be counted twice), but slightly lower number is expected since cell divisions are not counted as segmentation errors.

2.3 Choosing the correct value of the suppressed minima in h-minima transform

Since the watershed method is known to be prone to over segmentation, we preprocess the images with the h-minima transform to suppress all local minima smaller than the critical value. This critical value is chosen by visual inspection, but we check for small variations around this value. We find that, with the critical value of suppressed minima being 35, the segmentation procedure finds 374 cells more than with a critical value of 40. If critical value of suppressed minima is set to be 45, the segmentation procedure finds 272 cells less than with our chosen value of 40. The total statistic of recognized cells with a critical value of suppressed minima set to 40 is 17,850 cells. We compare the distribution of cell areas gained from those three different segmentation results and we find that those data sets differ significantly (Supplementary figure 2). For example, choosing 35 as a critical value of the suppressed minima leads to recognition of much smaller cells, and Kolmogorow-Smirnow-Test (KS test) rejects the null hypothesis that those cell areas come from the same distribution as the cell areas obtained with choosing 40 as a critical value of the suppressed minima. However, if we eliminate segmentation errors with our criteria (see section 3.1 in the manuscript), the K-S test fully accepts that those distributions are the same. It is our criteria that ensures elimination of all the segmentation errors (segmentation errors due to our image analysis methods and inherent errors due to relative positions of β-catenin and nuclei signal) in the set of data used for our comparison between morphological features of the cells' membranes and Voronoi tessellations based on the centers of mass of the nuclei (CMVT). Therefore, the data set is not sensitive to the small variations around chosen value of 40 for the critical value of the suppressed minima, because all the segmentation errors are eliminated by our criteria.

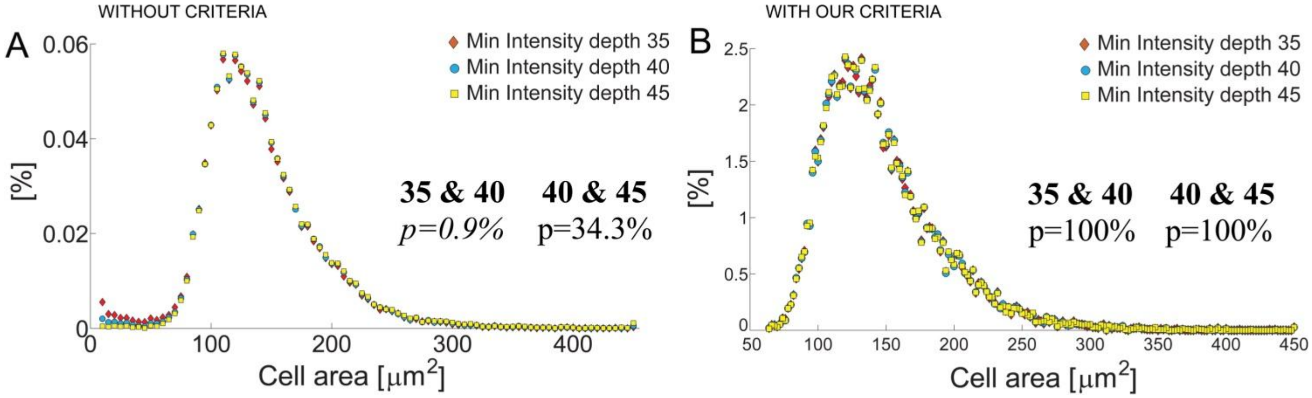

Supplementary Figure 2. Distributions of cell areas with three different choices for critical value of the suppressed minima in h-minima transform that precedes watershed based segmentation. A) Distribution of the areas without our elimination criteria leads to significant differences between the distributions. B) Distributions of areas after elimination of the segmentation errors with our criteria are not sensitive to the small variations around the critical value of the suppressed minima.

3 Segmentation procedure for MDCK II monolayer grown on hard PA gels

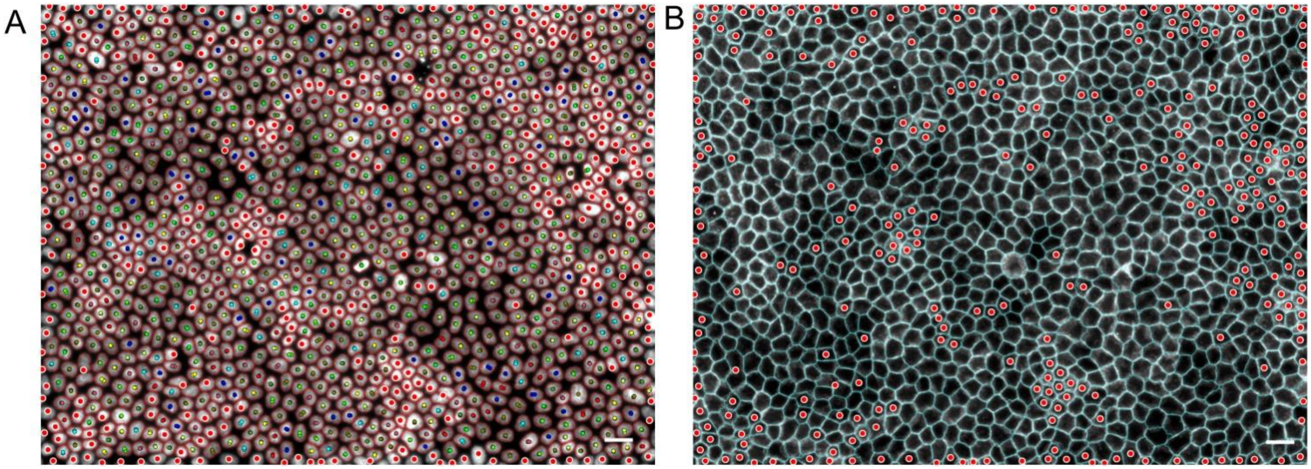

Supplementary Figure 3. Segmentation of the representative picture (out of 23 pictures) showing the whole 325.3x434.2 µm field of view. A) Segmentation of the cells' nuclei. Red outlines represent edges of the cells' nuclei. Big white-red dots mark the cells eliminated from presented data set because they did not satisfy the criteria. Small dots represent centers of mass of the cell nucleus and the corresponding cell membrane. Those cells enter the data set presented in the main text. B) Segmented membrane. White-red dots represent cells eliminated due to our criteria. Bars are 20 µm.

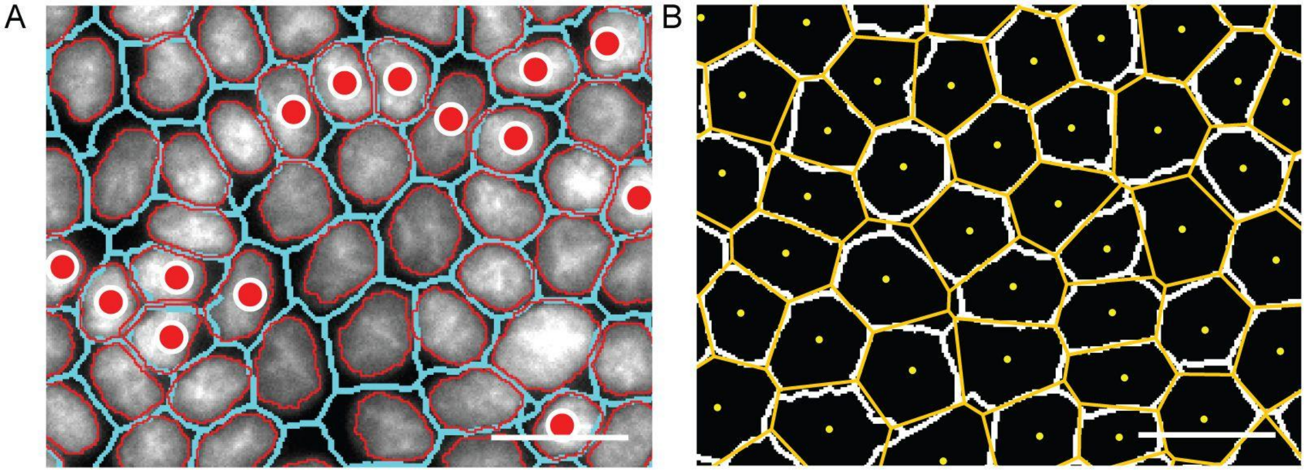

Supplementary Figure 4. Zoom in the regions of interest. A) Zoom in upper middle part of the representative picture. Red lines represent edges of segmented cell nuclei, and cyan lines represented segmented membrane. Big white-red dots are indicating cells eliminated from statistics due to the criteria that minimally 95% of the cell's nucleus belongs to the corresponding cell's membrane, which is not occupied by more than 5% of the neighboring nucleus. B) Zoom in down right corner of the representative picture where all the cells have satisfied the criteria. Yellow lines represent CMVT approximation of the real cell shape, while white lines represent the cell's membranes on the set of the cells that contribute to the data set analyzed in the paper. In total 15,000 of membranes have been compared with the corresponding CMVT in respect to six morphological measures. Bars are 20 µm.

4 Performance of CMVT on soft 0.6 kPa polyacrylamide (PA) gels

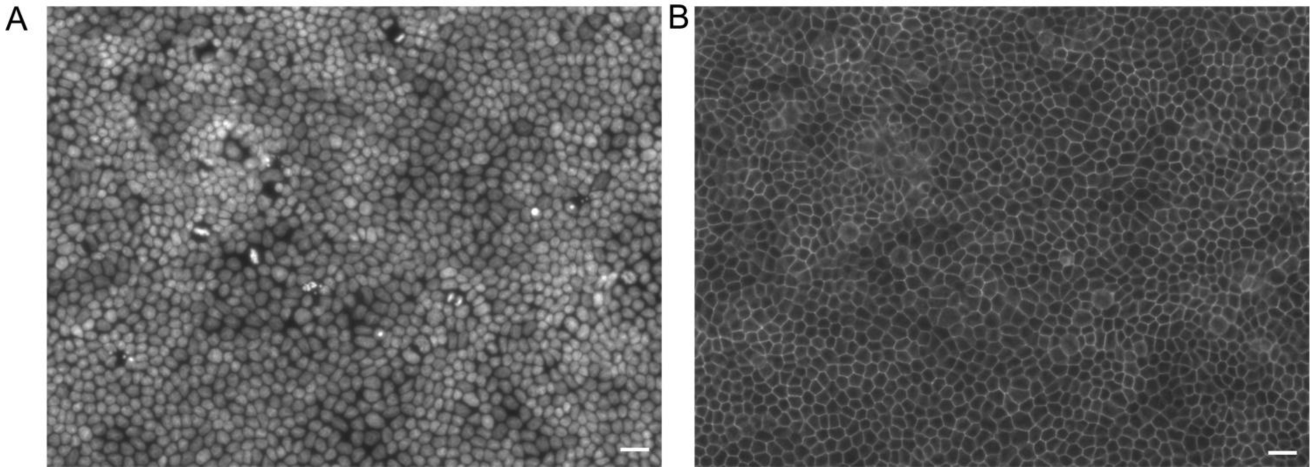

Supplementary Figure 5. Representative picture of MDCK II monolayer on soft (0.6 ± 0.2 kPa) PA gels. A) Hoechst stained cell nuclei. B) β-catenin stained cell membrane. Scale bars are 20 μm.

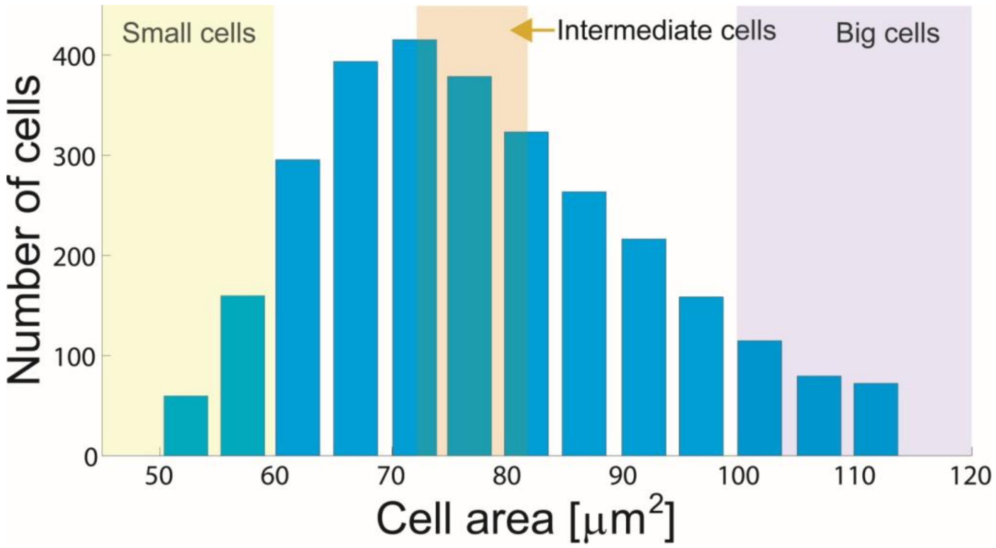

Supplementary Figure 6. Histogram of cell area of MDCK II on soft PA gels. Total number of cells is 3,097 while 2,937 cells belong to subsets. Average cell size is 80 ± 18 μm^2.

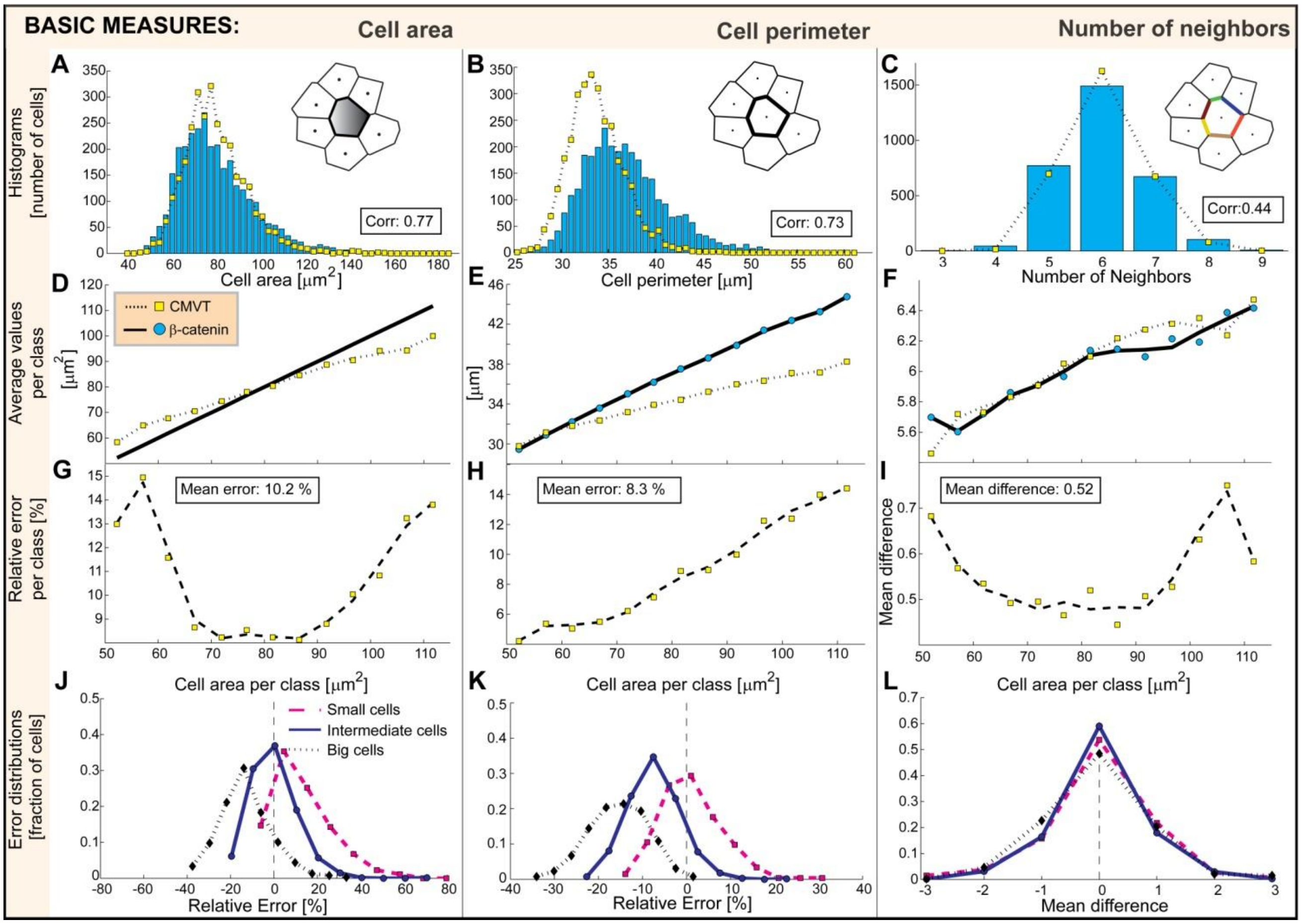

Supplementary Figure 7. Comparison of CMVT (yellow squares) and directly extracted morphological measures (blue circles). The graphs associated with cell areas, perimeters and number of neighbors are shown in the first, second and third column, respectively. Top graphs (A-C) show the probability distribution generated by direct measurement and estimated from the tessellation. (D-F) Second row is the average dependence of morphological characteristics on the cell area. The associated deviations of CMVT are shown in the third row (G-I). The distributions of errors for small, intermediate-sized and large cells are shown in the bottom row (J-L).

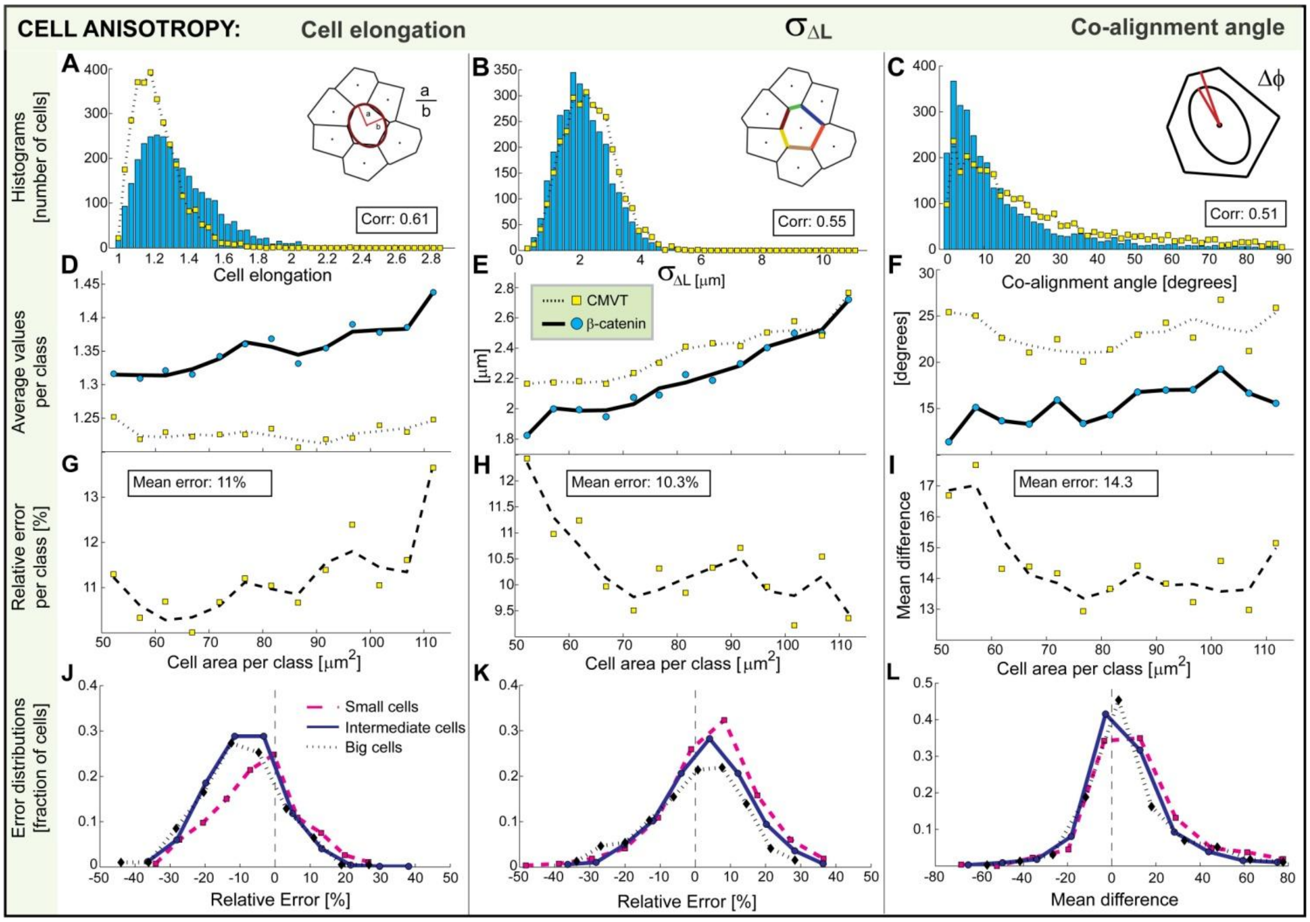

Supplementary Figure 8. Comparison of CMVT (yellow squares) and directly extracted morphological measures (blue circles). The graphs associated with cell elongation, standard deviation of boundary length and co-alignment of the nuclei and the cell are shown in the first, second and third column, respectively. Top graphs (A-C) show the probability distribution generated by direct measurement and estimated from the tessellation. (D-F) Second row is the average dependence of morphological characteristics on the cell area. The associated deviations of CMVT are shown in the third row (G-I). The distributions of errors for small, intermediate-sized and large cells are shown in the bottom row (J-L).

5 Performance of CMVT on glass substrate

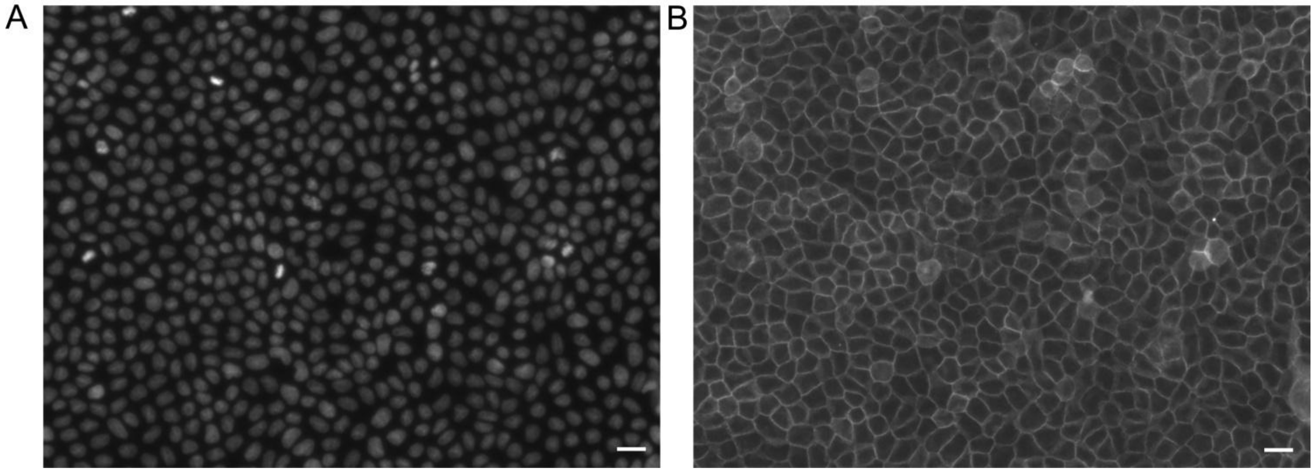

Supplementary Figure 9. Representative picture of MDCK II monolayer on glass substrates. A) Hoechst stained cell nuclei. B) β-catenin stained cell membrane. Scale bars are 20 µm.

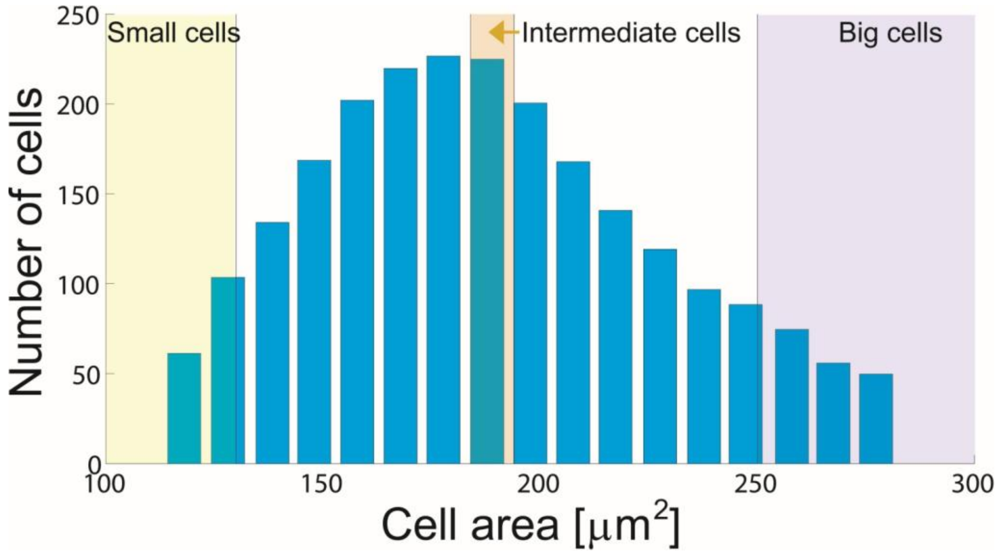

Supplementary Figure 10. Histogram of cell area of MDCK II on glass. Total number of cells is 2,576, while 2,335 cells belong to subsets. Average cell size is 196 ± 52 µm^2.

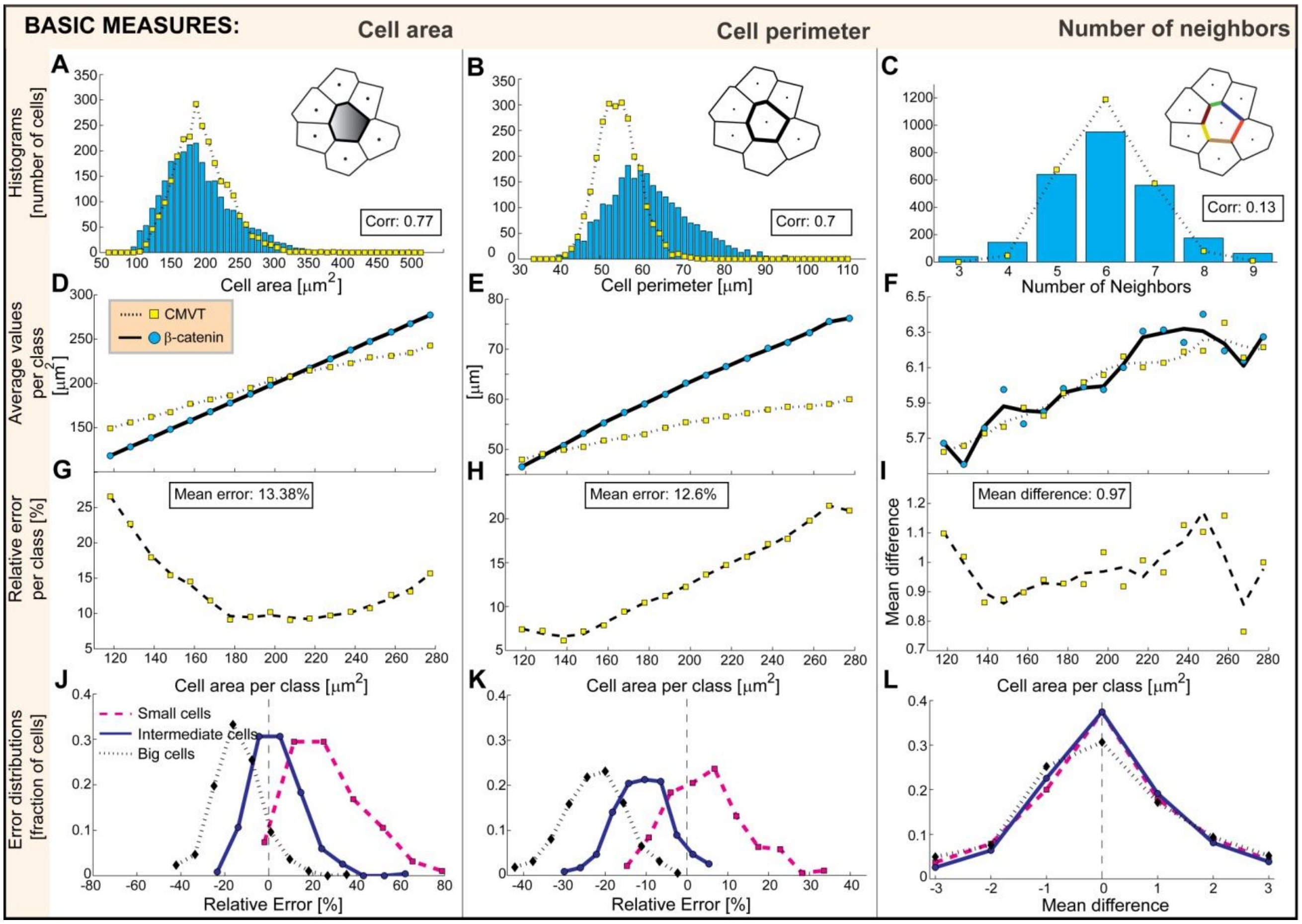

Supplementary Figure 11. Comparison of CMVT (yellow squares) and directly extracted morphological measures (blue circles). The graphs associated with cell areas, perimeters and number of neighbors are shown in the first, second and third column, respectively. Top graphs (A-C) show the probability distribution generated by direct measurement and estimated from the tessellation. (D-F) Second row is the average dependence of morphological characteristics on the cell area. The associated deviations of CMVT are shown in the third row (G-I). The distributions of errors for small, intermediate-sized and large cells are shown in the bottom row (J-L).

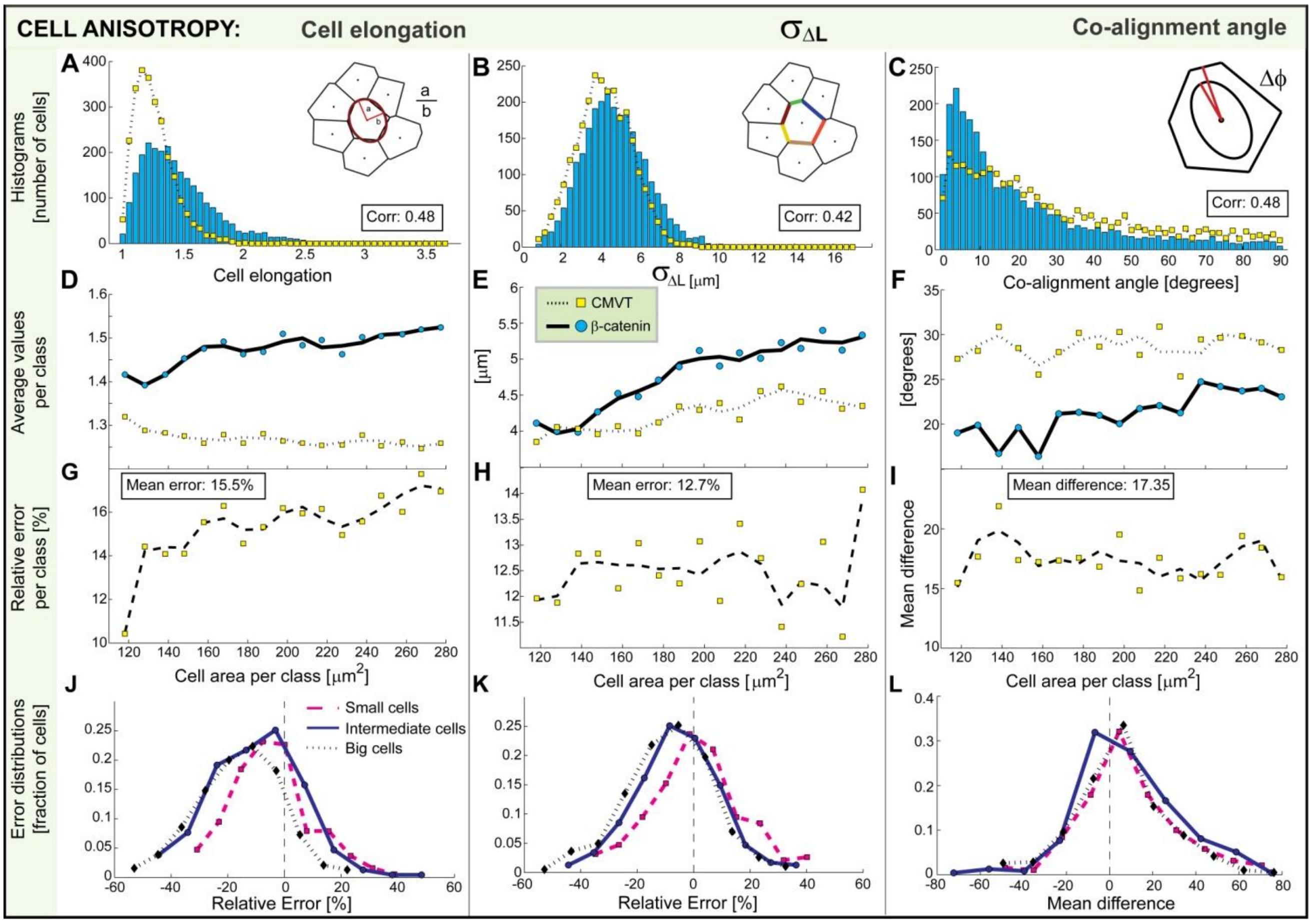

Supplementary Figure 12. Comparison of CMVT (yellow squares) and directly extracted morphological measures (blue circles). The graphs associated with cell elongation, standard deviation of boundary length and co-alignment of the nuclei and the cell are shown in the first, second and third column, respectively. Top graphs (A-C) show the probability distribution generated by direct measurement and estimated from the tessellation. (D-F) Second row is the average dependence of morphological characteristics on the cell area. The associated deviations of CMVT are shown in the third row (G-I). The distributions of errors for small, intermediate-sized and large cells are shown in the bottom row (J-L).

6 Scatter plots

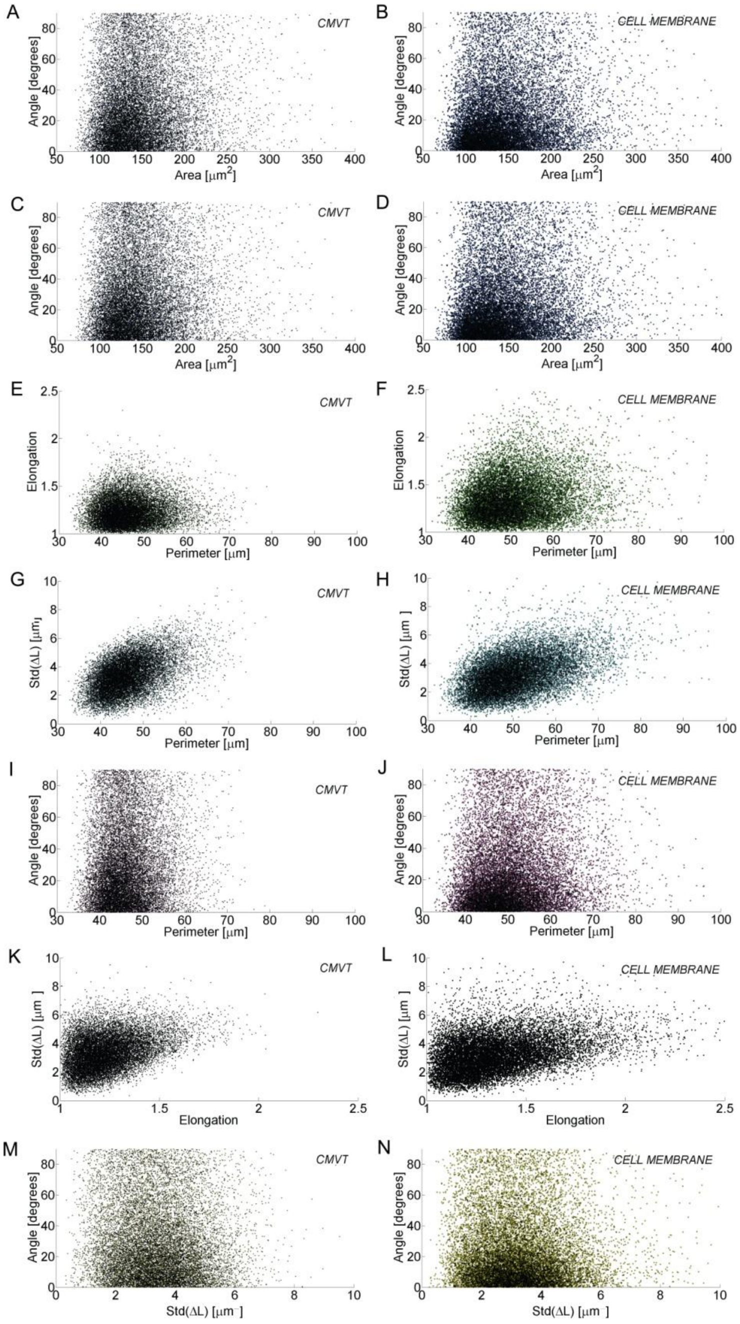

Figure 13. Scatter plots of the morphological measures.

7 Linear regression of the mean values

If we assume a linear dependence of the mean values of the morphological measures $\langle M_k^{CMVT} \rangle$ and $\langle M_k^{Memb} \rangle$ and the mean cell area in each subclass (Figures 6 and 7 in the main manuscript) we can write:

$$\langle \boldsymbol{M_k^{CMVT}} \rangle = \boldsymbol{k_{CMVT}} * \langle \boldsymbol{Area_k^{Memb}} \rangle + \boldsymbol{a_{CMVT}}$$

$$\langle \boldsymbol{M_k^{Memb}} \rangle = \boldsymbol{k_{Memb}} * \langle \boldsymbol{Area_k^{Memb}} \rangle + \boldsymbol{a_{Memb}}$$

We estimate $a_{CMVT}, k_{CMVT}, a_{Memb}$ and k_{Memb}, and calculate the scaling factors between values of the morphological measures estimated from the cells' membranes and values estimated by CMVT as:

$$\langle \boldsymbol{M_k^{Memb}} \rangle = \boldsymbol{A} * \langle \boldsymbol{M_k^{CMVT}} \rangle + \boldsymbol{B}$$

Table 5. Values of the scaling parameters between $\langle \boldsymbol{M_k^{Memb}} \rangle$ and $\langle \boldsymbol{M_k^{CMVT}} \rangle$.

	A	B
Area	1.292	−41.99
Perimeter	1.593	−23.05
Neighbors	1.158	−0.964
Elongation	−3.087	9.079
STD(ΔL)	1.228	−0.895
Angle	0.17	7.94

[1] http://imagej.net/Nuclei_Watershed_Separation. Accessed 20 Sep 2016.
[2] http://cellprofiler.org/manuals.html. Accessed 20 Sep 2016.
