# Supplementary figures and images for: Limits of Applicability of the Voronoi Tessellation Determined by Centers of Cell Nuclei to Epithelium Morphology
Source: Front Physiol. 2016 Nov 25;7:551. doi: 10.3389/fphys.2016.00551 (PMC5122581; doi:10.3389/fphys.2016.00551)

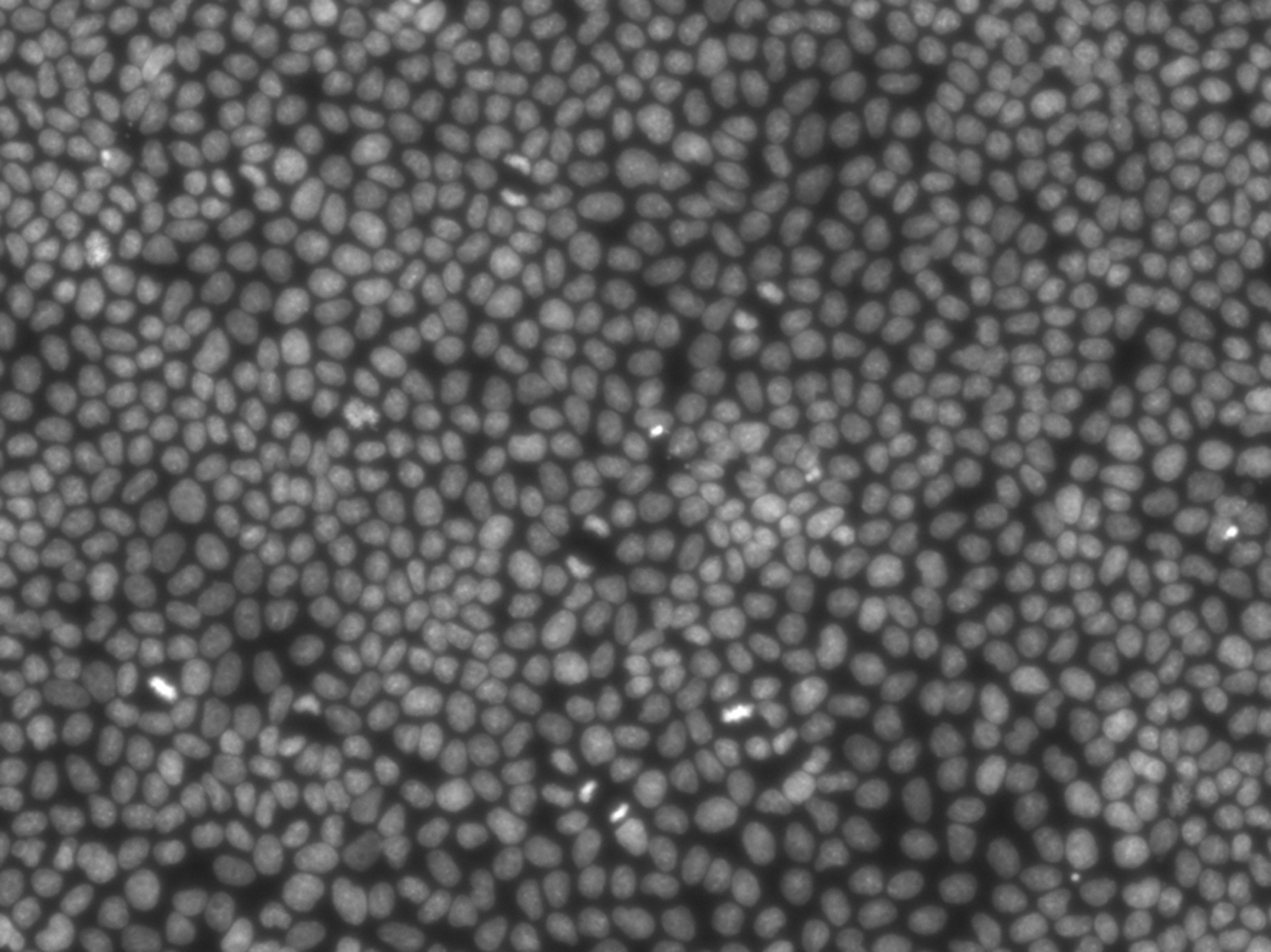

Supplement: Supplementary file 2 [file Image1.TIF]

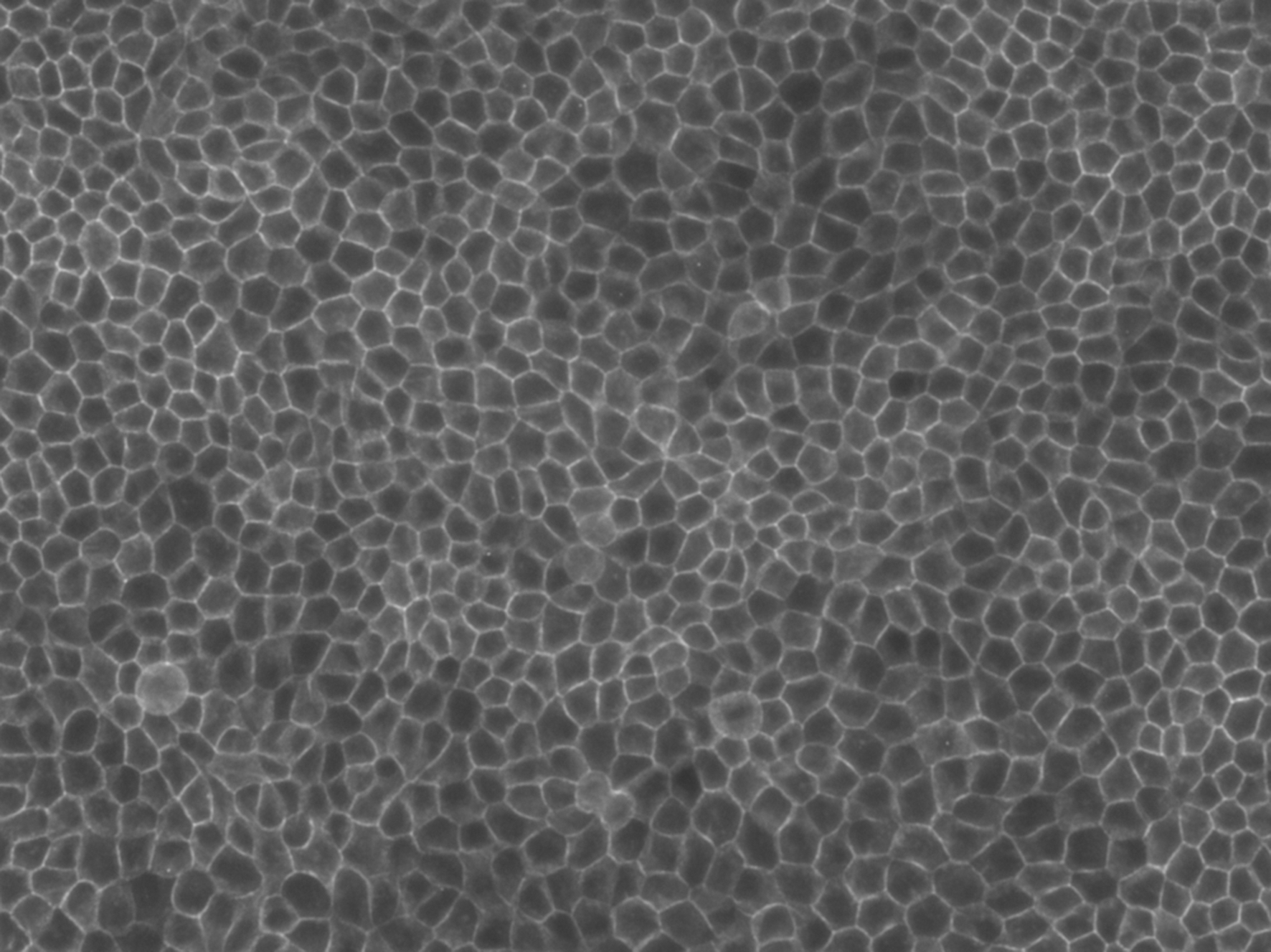

Supplement: Supplementary file 3 [file Image2.TIF]

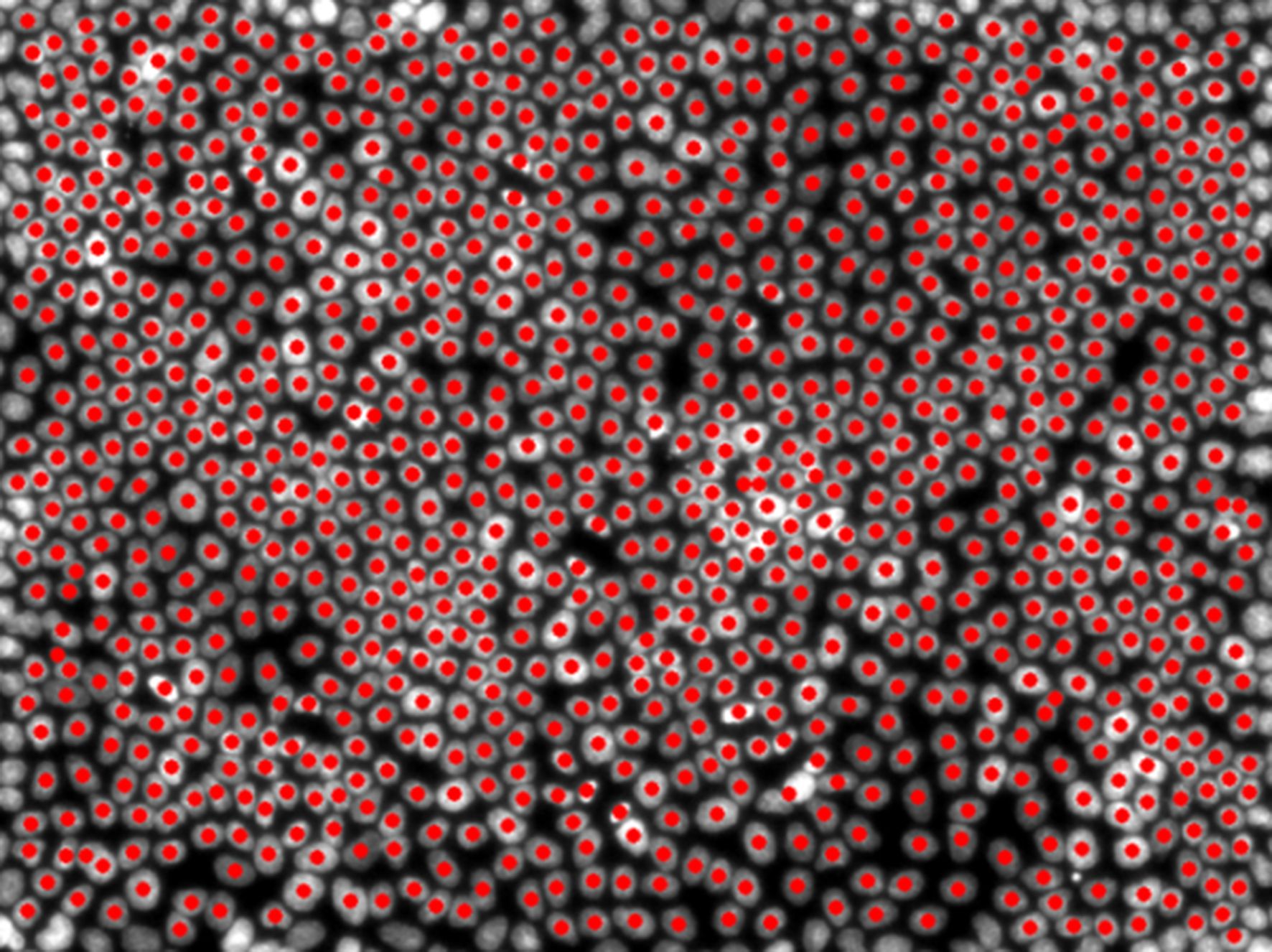

Supplement: Supplementary file 4 [file Image3.TIF]

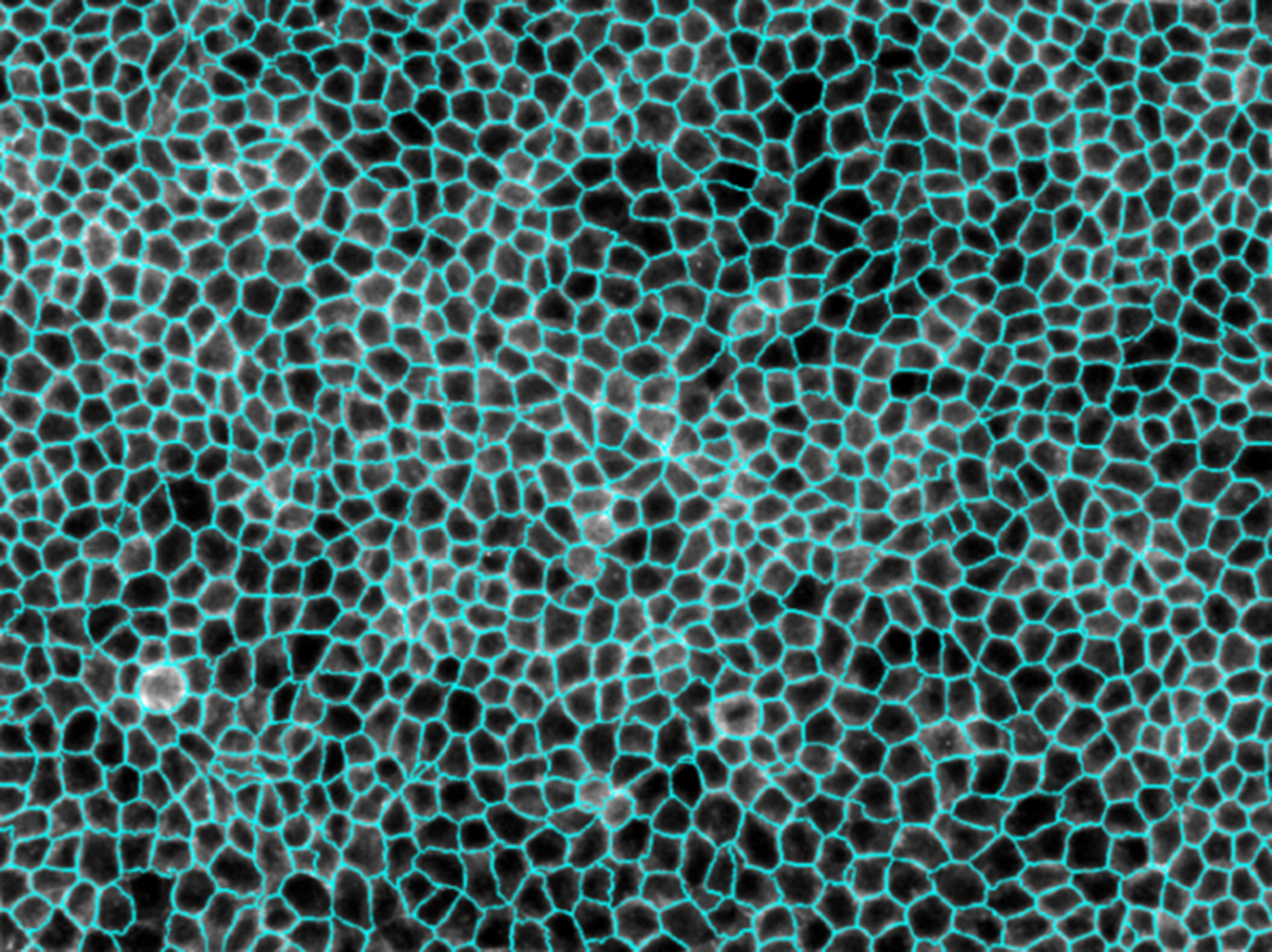

Supplement: Supplementary file 5 [file Image4.TIF]
